# Redox regulation of glutamate-1-semialdehyde aminotransferase modulates the synthesis of 5-aminolevulinic acid in Arabidopsis

**DOI:** 10.3389/fpls.2025.1645191

**Published:** 2025-11-21

**Authors:** Neha Sinha, Rana Hussein, Jerome Paul, Marc Nazare, Bernhard Grimm

**Affiliations:** 1 Humboldt-Universität zu Berlin, Institute of Biology/Plant Physiology, Berlin, Germany; 2 Humboldt-Universität zu Berlin, Institute of Biology/Structural Biology and Biochemistry, Berlin, Germany; 3 Leibniz-Forschungsinstitut für Molekulare Pharmakologie (FMP), AG Medizinische Chemie, Berlin, Germany

**Keywords:** tetrapyrrole biosynthesis, chlorophyll metabolism, redox control, thiol-disulfide switch, thioredoxin

## Abstract

Plants are constantly exposed to sudden changes in environmental parameters and must respond quickly to changes in temperature, humidity and light conditions. Such fluctuations in growth conditions also require almost immediate adjustments in the synthesis of photosynthetic pigments. Post-translational redox control of tetrapyrrole metabolism for chlorophyll and heme synthesis provides the necessary modifications for photosynthesis. The enzyme glutamate-1-semialdehyde aminotransferase (GSAAT) contributes to the rate-limiting step in the synthesis of 5-aminolevulinic acid (ALA). We intend to specifically investigate the redox control of GSAAT, analyze the redox-dependent shifts in the thiol-disulphide state of GSAAT, and identify the redox-dependent cysteines responsible for changes in the structure, enzymatic activity and stability of the protein. Wild-type GSAAT and Cys→Ser substitution mutants of the enzyme were examined for their activities with the labile substrate of GSAAT, glutamate-1-semialdehyde, which was synthesized in a simplified manner using a novel method. We show that of the four cysteine residues found in GSAAT, Cys168 and Cys190 are crucial for the redox-regulated state of GSAAT. Based on these experiments, we propose a redox-dependent structural modification of GSAAT that could lead to a decrease in the activity of the oxidized protein compared to the reduced enzyme.

## 1 Introduction

The plant tetrapyrrole biosynthesis (TBS) pathway is responsible for the synthesis of chlorophyll (Chl), heme, phytochromobilin, and siroheme, all of which are indispensable for plant viability (Tanaka et al., 2011; Brzezowski et al., 2015). Owing to the quantitatively diverse demands for these functionally distinct tetrapyrrole end-products, the metabolic pathway in plastids consists of at least 25 enzymatic reactions, which are tightly regulated at the transcriptional and post-translational levels (Mochizuki et al., 2010; Tanaka et al., 2011; Czarnecki and Grimm, 2012, Kobayashi and Masuda, 2016). Precise control of tetrapyrrole metabolism in higher plants is primarily required to avoid the accumulation of photoreactive intermediates and end-products that would otherwise lead to subcellular photo-oxidative damage and cell death. The control of the TBS pathway, and in particular the synthesis of chlorophyll (Chl), are tightly regulated by both light-dependent and thiol-switch-based redox mechanisms during the biogenesis and maintenance of functional chloroplasts. The redox status-dependent regulatory processes involve the reversible formation of disulfide bonds between the thiol groups of two cysteine (Cys) residues, within a given protein, between different proteins, or between a Cys and glutathione or sulfides. These processes lead to modulations of the activity, folding, and stability of plastid-localized TBS enzymes (Richter and Grimm, 2013; Dietz and Hell, 2015; Nikkanen et al., 2016).

In Arabidopsis, the isoforms of plastid-localized thioredoxins (TRXs, i.e., TRX-f1 and -f2, TRX-m1, -m2, -m3 and -m4, TRX-x, TRX-y1 and -y2 and TRX-z) together with the NADPH-dependent thioredoxin reductase (NTRC), provide a redox regulation system that enables rapid and reliable reduction of proteins during the transition from dark to light (Michalska et al., 2009; Michelet et al., 2013; Serrato et al., 2013; Balsera et al., 2014). It has been shown that deficiency of the f- and m-type TRX variants and NTRC results in multiple defects in the TBS pathway and leads to a pale-green leaf phenotype (Serrato et al., 2004; Lepistö et al., 2009; Wang et al., 2013). So far, five TBS enzymes have been shown to serve as targets for TRX- and NTRC-mediated reduction of thiol bonds: glutamyl-tRNA reductase (GluTR), the rate-limiting enzyme in 5-aminolevulinic acid (ALA) synthesis at the onset of the TBS pathway, 5-aminolevulinic acid dehydratase (ALAD) and members of the Chl synthesis branch, i.e., subunit I of magnesium chelatase (CHLI), magnesium protoporphyrin IX methyltransferase (CHLM) and Mg protoporphyrin monomethylester cyclase (CHL27) (Richter et al., 2013; 2018; Ikegami et al., 2007; Luo et al., 2012; Stenbaek and Jensen, 2010; Wittmann et al., 2018, 2020). Apart from these enzymes, which have already been individually investigated for redox control and thiol switches, TBS enzymes such as glutamate 1-semialdehyde aminotransferase (GSAAT), GluTR-binding protein (GBP), porphobilinogen deaminase (PBGD), uroporphyrinogen III synthase (UROS), uroporphyrinogen III decarboxylase (UROD), coproporphyrinogen-oxidase (CPO), GENOMES UNCOUPLED 4 (GUN4), and protochlorophyllide oxidoreductase (POR) have been pinpointed as potentially interacting partners of TRXs and NTRC (Balmer et al., 2003; Marchand et al., 2006; Perez-Perez et al., 2017; González et al., 2019; Wittmann et al., 2020).

**Figure 1:**
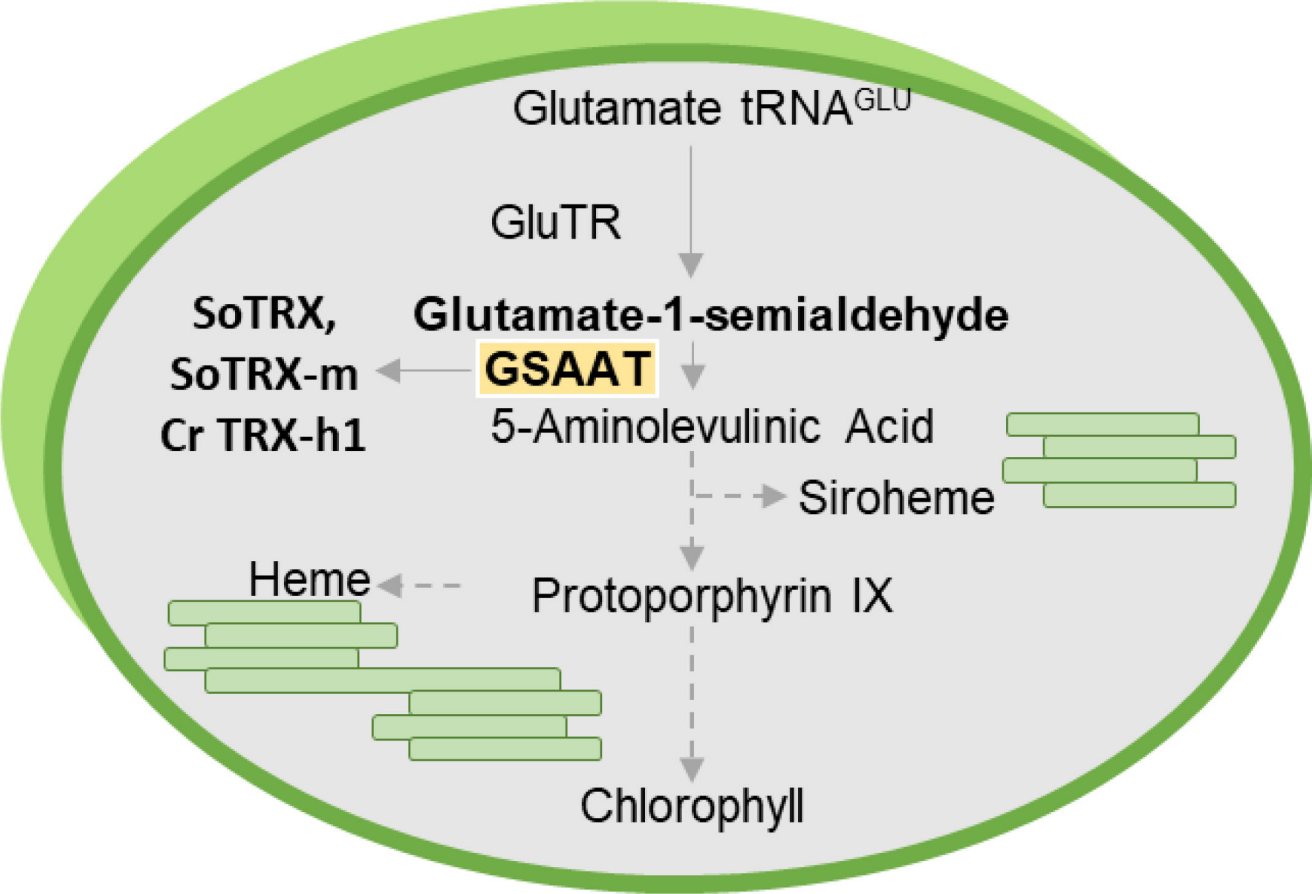
Schematic diagram of plant tetrapyrrole biosynthesis, which is responsible for the production of Chl, heme and siroheme. The emphasis here is on glutamate-1-semialdehyde aminotransferase (GSAAT), which is targeted by the redox regulators TRX-f and TRX-m in *Spinacia oleracea* (*So*) and TRX-h1 in *Chlamydomonas reinhardtii (Cr)*.

GSAAT catalyzes the intramolecular transfer of an amino group from glutamate-1-semialdehyde (GSA) to ALA (**Figure 1**). Two genes in the Arabidopsis genome encode isoforms of GSAAT: *GSA1* (At5G63570) and *GSA2* (At3G48730). In angiosperms, ALA synthesis is tightly controlled during photoperiodic growth: it is suppressed in darkness, and light-induced reactivated in a light-intensity dependent manner (Goslings et al., 2004; Hou et al., 2019). In addition to the light- and circadian-clock-induced transcriptional control of genes involved in ALA and Chl synthesis (Ilag et al., 1994; Matsumoto et al., 2004; Kobayashi and Masuda, 2016), post-translational modifications of TBS enzymes are essential for adequate synthesis of end-products and for the suppression of photoreactive metabolic intermediates (Wang et al., 2022). For example, post-translational inactivation of GluTR by the protein FLUORESCENT (FLU), which occurs in response to the accumulation of protochlorophyllide (PChlide) bound to protochlorophyllide reductase (POR), is responsible for the strictly controlled metabolic flow of tetrapyrrole intermediates during both light and dark growth phases (Meskauskiene et al., 2001). In addition, GluTR-binding protein (GBP) binds and stabilizes GluTR in the absence of heme, and releases the enzyme upon binding of heme, at which point it is targeted for proteolysis (Richter et al., 2018). However, it cannot be excluded that additional fine-tuning of ALA synthesis occurs at the post-translational level via a thiol-based mechanism, so as to rapidly adjust the synthesis rate to the demands for the end-products Chl and heme and the light-dependent activity of POR.

Previous redox- or TRX-based proteomic analyses have identified GSAAT as an interaction partner for TRXs or NTRC in *Chlamydomonas*, *Brassica*, *Spinacia oleracea* and *Arabidopsis thaliana* (Balmer et al., 2003; Zaffagnini et al., 2011, Zhu et al., 2014; Akter et al., 2015; Perez-Perez et al., 2017). It has also been shown that the isoforms spinach TRX-f and TRX-m and Chlamydomonas TRX-h1 interact with GSAAT (Balmer et al., 2003; Marchand et al., 2006; Pérez-Pérez et al., 2017). In the Arabidopsis triple mutant *trxm1/m2/m4* and the *trxf1/ntrc* mutant line, the stability of GSAAT is reduced (Da et al., 2017; Wittmann et al., 2018). In *C. reinhardtii*, the TRX-targeted cysteines in GSAAT were identified among the five Cys residues 82, 157, 179, 287 and 404 by means of TRX-affinity chromatography (Perez-Perez et al., 2017). These studies point to GSAAT as a redox-regulated enzyme. Nevertheless, the question remains as to how TRXs control its stability and activity, thereby contributing to redox-dependent ALA synthesis. We set out to examine the effects of thiol-based control on the stability and activity of the *A. thaliana* GSAAT (GSAAT_At_), and determine which of the several Cys residues in GSAAT are specifically involved in redox-dependent dithiol-disulfide transitions.

## 2 Methodology

### 2.1 Plant materials and growth conditions

The *Arabidopsis thaliana* T‐DNA insertion mutants *gsa2* (GABI_364C09), *ntrc* (SALK_012208), *trxf1* (SALK_128365) and *ntrc/trxf1* were grown under short-day conditions (SD; 10 h light/14 h dark) and standard light intensity (110 μmol photons m^−2^ s^−1^) at 22°C.

### 2.2 Cloning and mutagenesis of GSAAT for complementation of the *gsa2* knockout line

To construct the *35S::GSAAT* Cys substitution mutants, the At*GSA1* gene was amplified using appropriate primers (**Supplementary Table 1**) from Arabidopsis Col-0 genomic DNA, cloned into the entry vector pJet1.2 (Thermo Scientific) and used as a template for site-directed mutagenesis in which single cysteine codons were replaced by gene-specific serine codons (**Supplementary Table 1**) as suggested by Liable and Boonrod, 2009. The resulting constructs, i.e. GSAAT(C138S), GSAAT(C168S), GSAAT(C190S), GSAAT(C396S), and the GSAAT(WT) control were then transformed into *gsa2* mutants using the plant transformation vector pCAMBIA-Strep (driven by a 35S promotor) and the *Agrobacterium tumefaciens* strain GV2260. These Arabidopsis seedlings were grown on soil under SD conditions for further analyses.

### 2.3 Purification of recombinant His-GSAAT, His-TRX f1, His-TRX m1 and His-NTRC proteins

The cDNA sequence for the mature *AtGSA1* (AT5G63570, without the transit peptide sequence 1–38 for plastid targeting, according to https://www.uniprot.org/uniprotkb/P42799/entry), was cloned into the pET-28a(+) vector, which includes an N-terminal 6X histidine (His)-tag (Novagen, Merck Millipore, Burlington, MA, USA). These constructs were then transformed into *E. coli* Rosetta cells, and their expression was induced by adding 0.4 mM isopropyl-D-1-thiogalactopyranoside (IPTG) at 16°C for 14–16 h. His-tagged TRX f1, TRX m1, and NTRC were expressed similarly and induced with IPTG for 3 h at 37°C. All the proteins were purified using nickel-nitrilotriacetic acid (Ni-NTA) resin (Thermo Fisher Scientific, Waltham, MA, USA)) according to the manufacturer’s protocol. Finally, the purified proteins were dialyzed and concentrated using Amicon^®^ Ultra-4 Centrifugal Filter Units (10K/30K) (Merck-Millipore, Burlington, MA, USA).

### 2.4 Protein extraction, alkylation assays and immunoblot analyses

Three-week-old leaf tissue (20–30 mg) was frozen in liquid nitrogen, and homogenized. The samples were dissolved in 200-300 μl of 2×SDS‐PAGE sample buffer (100 mM Tris/HCl pH 6.8, 4% SDS, 20% glycerol and 2 mM DTT), denatured at 95°C for 5 min, and centrifuged for 1 min at 16,000 g at room temperature.

*In-vivo* alkylation assays were performed as previously described (Naranjo et al, 2016). Aliquots (25–30 mg) of leaf tissues were frozen in liquid nitrogen and homogenized directly in 300 µl of 10% (v/v) trichloroacetic acid (TCA) to prevent any oxidation/reduction. The samples were incubated on ice for 30 min and then centrifuged for 10 min at 16,000 g at 4°C. The pellets were then washed twice with 500 µl of acetone for 10 min each, centrifuged at 16,000 g at 4°C, resuspended in alkylation buffer (2% SDS, 50 mM TRIS–HCl pH 7.8, 2.5% glycerol, and 4 M urea), and incubated with either 10 mM methyl-maleimide polyethylene glycol (MM-PEG_50_), 60 mM IAA (iodoacetamide) or 1 mM AMS (4-acetoamido-4-maleimidylstilbene-2,2-disulfonic acid) for 30 min at room temperature to alkylate protein thiols.

For *in-vitro* alkylation assays, 200–300 ng of purified recombinant His-tagged GSAAT proteins were pre-incubated with diamide, CuCl_2_ or DTT in PBS buffer (150 mM NaCl, 20 mM Na_2_HPO_4_, pH 7.4) for 15 min at room temperature. Subsequently, the proteins were precipitated using TCA as described previously, and incubated with 1-3 µM TRXf1/TRXm1 or 100 mM N-ethylmaleimide (NEM) as indicated (Wittmann et al., 2018). The proteins to be treated with NEM were completely reduced with 100 mM DTT prior to TCA precipitation. Finally, the precipitated proteins were incubated with MM-PEG_50_ to label the modified cysteines. Aliquots (10‐15 μl) of each sample were then loaded onto 10% or 12% reducing or non-reducing SDS PA-gels for subsequent immunoblot analysis using specific antibodies.

### 2.5 Preparation of glutamate 1-semialdehyde/4-amino-5-oxopentanoic acid

Tert-Butyl 4-{[(tert-butoxy)carbonyl]amino}-5-oxopentanoat (100 mg, 0.348 mmol) was dissolved in dry dichloromethane (2.5 ml). To this solution 1.5 ml 4N HCl in dioxane were added at room temperature. The mixture was stirred for 8h at room temperature. Thereafter the reaction mixture was diluted with 10 ml of diethylether. After sonication for 5 min, n-pentane (10 ml) was added and sonication was repeated for 5 min. After standing for 10 min, the supernatant was removed. The solid residue was mixed again with 5 ml of diethylether and sonicated for 5 min. After standing for 10 min, the supernatant was removed and the remaining solid was dried in high vacuum to yield glutamate 1-semialdehyde (4-amino-5-oxopentanoic acid) as its hydrochloride salt. The yield was 56mg (96%). Purity and identity were confirmed by LC-MS (pos. ESI-MS): *m/z* calculated for C_5_H_9_NNO_3_ [M+H]^+^ 132.13, found 130.0 (**Supplementary Figure 4**). Mass spectra were recorded with an Agilent 1260 infinity liquid chromatography coupled quadrupole mass spectrometer 6120 detector.

### 2.6 Assay of GSAAT activity of recombinant proteins and plant extracts

In-planta GSAAT assays were performed as described previously (Hoober et al., 1988) with some modifications. The crude extracts for the enzyme assay were prepared by homogenizing the tissue in 0.1 M MOPS buffer (Na 2-(N-morpholino) ethanesulfonate-0.1 M Na phosphate) pH 6.8. Aliquots of the extract (200-300 µg) were pre-incubated with DTT or diamide or left untreated (UT) for 15 min at RT, then combined with 10-30 µM GSA, 10 µM pyridoxal phosphate (PLP) and 10 mM levulinic acid in a total volume of 1 ml, and incubated at 28°C for 10 min. The reaction was terminated by the addition of ethyl acetoacetate and adjusted to pH 6.8, followed by heating for 10 min at 100°C. The tubes were then cooled to room temperature and 1 volume of modified Ehrlich’s reagent (12.6% perchloric acid, 74.6% acetic acid, 11.4% HgCl_2_ and 0.4% 4-NN-dimethylamino)benzaldehyde) was added, and finally absorption was recorded at 553 and 526 nm as described by Mauzerall and Granick (1956).

*In-vitro* GSAAT assays were performed with 5 µg of His-GSAAT, pre-incubated with either DTT or diamide, or left untreated (UT), prior to the addition of 1-3 µM His-TRX f1 or His-TRX m1 for 10 min at RT. Further steps in the assay were carried out as described above.

### 2.7 Bimolecular fluorescence complementation assay

Full-length cDNA copies of Arabidopsis GSA, TRXf1 and NTRC genes were cloned into the pJET2.1 vector (Thermo Scientific) using appropriate primers (**Supplementary Table 1**). They were then fused with either the N-terminal or the C-terminal half of the YFP protein-containing plasmids pVyNE and pVyCE (Gehl et al., 2009; Invitrogen, Carlsbad, CA, USA), respectively. These fused plasmids were then transiently co-expressed by infiltration into tobacco (*Nicotiana benthamiana*) leaves via *Agrobacterium tumefaciens* GV2260. The tobacco leaf discs with the expressed proteins were then analyzed for yellow fluorescent signals after 2 days of dark incubation using an LSM 800 confocal microscope (Zeiss; λex 514 nm, λem (YFP) 530–555 nm, λem (Chl) 600–700 nm).

### 2.8 Pull-down experiments

Chloroplast extracts (100 µg of Chl) were solubilized with 1% (w/v) dodecyl maltoside (DM) for 10 min at 4°C, and incubated with 50 μg of either purified His-TRX-f1, His-TRX-m1 and His-NTRC as bait proteins in binding buffer (BF, 25 mM Tris-HCl [pH 7.8], 150 mM NaCl, 5 mM MgCl_2_, 10% [v/v], glycerol, and cOmplete protease inhibitor [Roche]) overnight at 4°C and 45 rpm. Then 50 μl of Ni-NTA agarose (Thermo Fisher Scientific) was added to each extract containing His-GSAAT proteins, and incubated for 2 h, as before. Ni-NTA resin-bound proteins were washed six times by centrifugation at 3,000 rpm for 5 min each at 4°C using BF supplemented with 10 mM imidazole. Finally, the Ni-NTA resin-bound proteins were eluted with BF + 200 mM imidazole, fractionated on a reducing 12% SDS-PA gel and probed with TRX f1 and NTRC antibodies following immunoblotting.

### 2.9 Transcriptional analysis by qRT-PCR

cDNAs were synthesized from 2 μg RNA pretreated with DNase I (Thermo Scientific) as described in Sinha et al. (2022). qRT-PCR primers used in this study are listed in **Supplementary Table 1**.

## 3 Results

### 3.1 Structural analysis and protein sequence alignments reveal four highly conserved cysteine residues in Arabidopsis GSAAT

A X-ray crystallographic structure of Arabidopsis GSAAT1 (GSAAT_At_) has been reported at 1.25 A˚ resolution (Song et al., 2016). In agreement with a previous structure for *Synechocystis* GSAAT (GSAAT_Syn_), GSAAT_At_ forms an asymmetric dimer, which reflects the differential binding of its substrates pyridoxal 5’-phosphate (PLP) and pyridoxamine 5’-phosphate (PMP) as cofactors to the two subunits, respectively (Hennig et al., 1997; Song et al., 2016). The transit peptide is 40 amino acid residues (aa) long, while the mature AtGSAAT1 protein is comprised of 474 aa. The large catalytic pocket is made up of between residues 104 (Tyr) and 368 (Gly), which are flanked by a 63-aa N-terminal domain and a 106-aa C-terminal segment. The lysine residue K274 of the mature enzyme (also designated K314 in the sequence of the GSAAT_At_ precursor) is located close to the bound cofactor PLP, with which it forms a Schiff-base linkage (Hennig et al., 1997; Stetefeld et al., 2006; Song et al., 2016).

GSAAT_At_ has four conserved Cys residues, Cys138, Cys168, Cys190 (all present in the catalytic domain) and Cys396 (at the C-terminus). BLAST searches were carried out on the NCBI website (http://blast.ncbi.nlm.nih.gov/Blast.cgi) and sequence alignment of GSAATs from different species was performed using MUSCLE (https://www.ebi.ac.uk/Tools/msa/muscle/) and visualized using ESPript software (https://espript.ibcp.fr/ESPript/ESPript/) (**Supplementary Figure 1**). All four cysteine residues are conserved in higher plants, the first three Cys residues (Cys138, Cys168, Cys190) are conserved in the single *C. reinhardtii* GSAAT, whereas only Cys190 is retained in the GSAAT of *Chlorobium*, a genus of green sulfur bacteria. In summary, as conserved cysteine residues are potential candidates for the redox control of proteins due to their ability to undergo reversible oxidation-reduction reactions, participate in enzyme catalysis, maintain structural integrity, and sense the cellular redox environment, Cys190 conservation across different species underscores its essential role in GSAAT enzyme catalysis or its regulatory function (**Supplementary Figure 1**).

### 3.2 Redox-dependent modifications of the structure and activity of recombinant Arabidopsis GSAAT

**Figure 2:**
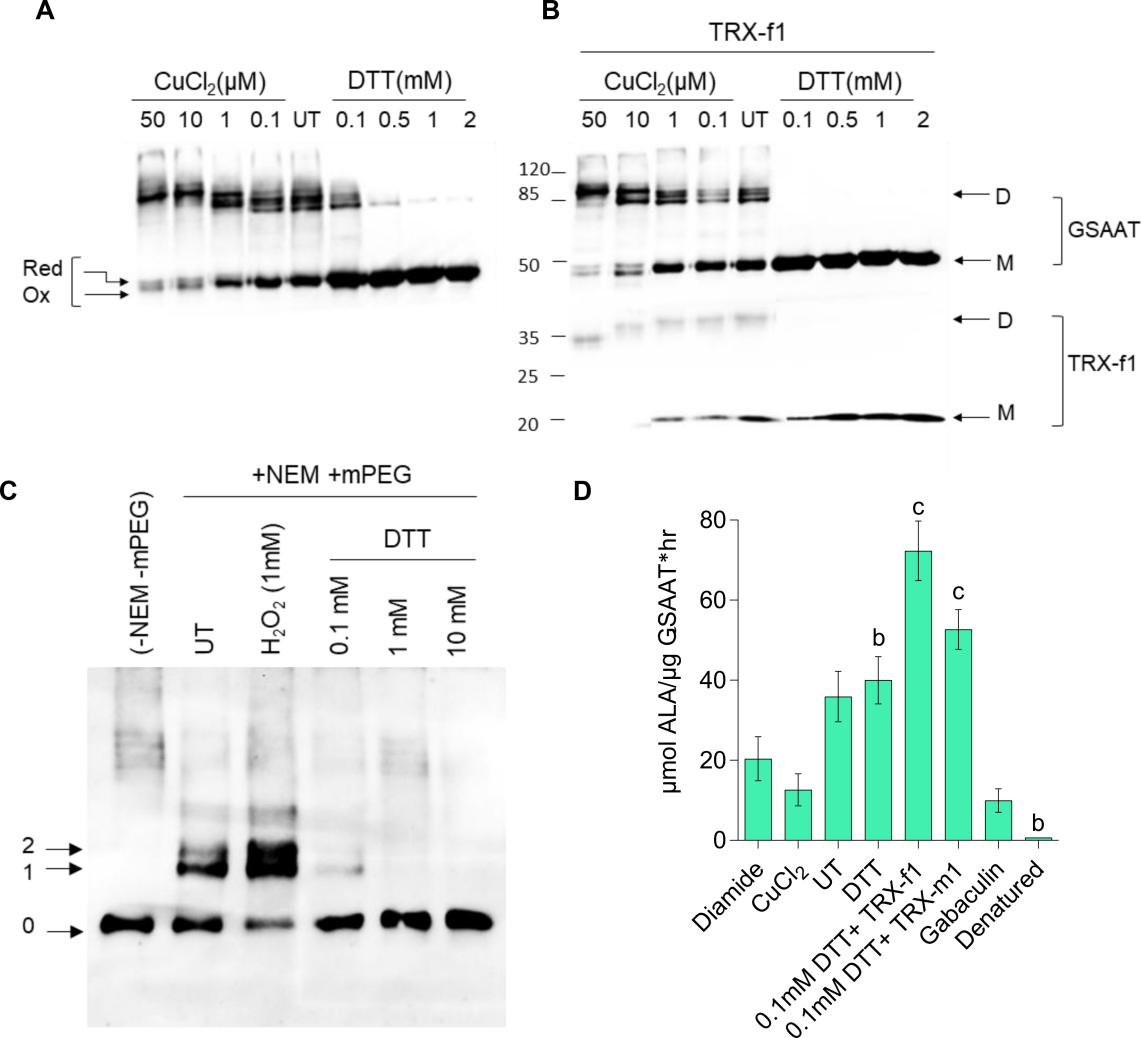
Redox-dependence of the structure and activity of the purified recombinant 6xHis-GSAAT. **(A)** Formation of monomeric and dimeric GSAAT, and depiction of its redox state under indicated oxidized (CuCl_2_), untreated (UT), and reduced (DTT) conditions. **(B)** Results of the same experiment performed in the absence and presence of 3 µM TRX-f1. The arrows in **(A)** indicate the different redox states of the GSAAT monomer (reduced, (red) and oxidized (ox)) triggered by changes in the protein’s mobility after the formation of internal disulfide bonds. In **(B),** the arrows indicate the monomer (M) and dimer **(D)** forms of GSAAT, and the enzyme TRX-f1. **(C)** Labeling of oxidized and buried cysteines with methoxypolyethylene glycol maleimide (mPEG-MAL)-5000. After pretreatment with either an oxidizing compound (1 mM hydrogen peroxide) or various concentrations of the reducing agent DTT, all exposed cysteines were irreversibly blocked by reaction with N-ethylmaleimide (NEM). Subsequently, all samples were reduced with DTT (100 mM) and labeled with mPEG-MAL-5000. The arrows labeled 0–2 indicate the unlabeled and the reduced and oxidized forms of labeled GSAAT, respectively. All the protein samples from **(A–C)** were fractionated on a non-reducing 10% SDS-polyacrylamide gel (PA; UT: untreated) and visualized with a His-tag-specific antibody. **(D)** GSAAT activity assay after pre-incubation of recombinant GSAAT (30–50 nM) with either oxidizing agents (200 µM diamide, 50µM CuCl_2_), reducing agents (2mM DTT, 3uM recombinant TRX-f1 or TRX-m1), or the GSAAT inhibitor gabaculine. UT indicates untreated samples. The amount of ALA formed was photometrically measured using Ehrlich’s reagent. Error bars represent SD of three technical replicates (three different assay reactions). Letters above histograms indicate significant differences as determined by using Student’s t test, where a is P ≤ 0.05, b is P ≤ 0.01, and c is P ≤ 0.001. The statistical significance was performed between the UT GSAAT compared to oxidized, reduced, denatured and gabaculine-treated GSAAT protein.

Fractionation of purified recombinant His-GSAAT_At_ (300 µM) expressed in *E. coli* on a non-reducing/non-denaturing polyacrylamide (PA) gel revealed that approximately half of the protein migrated as a dimer and the other half as a monomer (**Figure 2A**). Separation of proteins on a non-reducing SDS-PA gel, after addition of increasing amounts of the oxidizing agent copper chloride (CuCl_2)_ to GSAAT resulted in the predominance of the dimeric form, while GSAAT progressively reverted to the monomeric form as the content of the reducing agent dithiothreitol (DTT) was increased. Concentrations exceeding 0.5 mM DTT completely converted GSAAT into the monomeric form. GSAAT has been identified as a potential target of the reductants TRX-f and TRX-m in spinach chloroplasts (Balmer et al., 2003). Indeed, the disulfide bonds of GSAAT_At_ were also reduced when TRXs were added to the purified protein, thus preventing the potential formation of intra- and intermolecular disulfide linkages. The addition of 3 µM purified His-tagged TRX-f1 to His-GSAAT_At_ after pre-incubation with either an oxidant (CuCl_2_) or a reductant (DTT) promoted the formation of reduced monomeric GSAAT. The GSAAT dimer was completely converted into the monomeric form at a concentration of 0.1 mM added DTT upon recycling of the oxidized TRX isoform (**Figure 2B**). Incubation of GSAAT_At_ with 5 µM TRX-m1 (plus 0.1 mM DTT), following the separation on a non-reducing SDS PA gel, still resulted in a residual amount of GSAAT dimers (**Supplementary Figure 2**). This finding suggests that the *in vitro* reducing capacity of TRX-m1 on GSAAT is lower than that of TRX-f1.

It can be assumed that GSAAT_At_ is able to switch between various reduced thiol groups of Cys residues and oxidized disulfide bond(s) involving Cys residues, glutathione or sulfides. To assess the number of redox-sensitive Cys residues in GSAAT, the enzyme was treated with hydrogen peroxide or DTT (0.1–10 mM DTT) and subsequently with N-ethylmaleimide (NEM) to irreversibly block the free thiol groups of Cys residues in GSAAT. Then, after incubation with 100 mM DTT, GSAAT was treated with methoxypolyethylene glycol maleimide 5000 (mPEG maleimide5000). mPEG-MAL binds to every free Cys and alters the mobility of the protein on a non-reducing SDS-PA gel electrophoresis (PAGE) depending on how many thiol-mPEG conjugates are formed. Two additional immunoreactive GSAAT bands were observed. This can be interpreted that two Cys residues were oxidized, so that they could not react with NEM. But it is not excluded that also three (or four) Cys residues could be labeled with methoxypolyethylene glycol maleimide (mPEG-MAL)-5000, due to the broader difference in molecular mass of the immune-reacting GSAAT-band with zero (0) or one (1) cysteine bound to mPED-MAL. This assessment is even more relevant if it is considered that the oxidized state would lead to an intramolecular disulfide bond, which usually could not lead to a single reduced Cys residue under reducing conditions, but must allow two bonds with mPEG-MAL, unless the second Cys residue is structurally or spatially hidden. Indeed, when GSAAT is reduced with gradually increasing amounts of DTT prior to incubation with NEM, either one Cys (Band 1, with 0.1 mM DTT) or no Cys residue of GSAAT is accessible for mPEG binding (with DTT ≥1 mM) (**Figure 2C**). Consequently, with increasing amounts of added DTT, GSAAT becomes more accessible to NEM prior to treatment with mPEG-MAL. We assume that at least two Cys residues of GSSAT are oxidized, which may form an intracellular disulfide bond under oxidizing conditions. The reasons for the detection of bands 1 and 2 in the oxidized state will be further discussed in the Discussion section.

His-GSAAT_At_ should also undergo redox-dependent structural alterations that may affect its enzymatic activity. The GSAAT activity was therefore examined under oxidized (diamide, CuCl_2_) and reduced conditions (DTT and TRX’s). Oxidized GSAAT has a lower enzyme activity than GSAAT in the absence of any pretreatment (UT = untreated). Compared to the UT proteins, the dependence of DTT on His-GSAAT was determined, where the GSAAT activity was not considerably stimulated in the presence of DTT. Moreover, the GSAAT activity was activated by 2-fold when pre-treated with His-TRXf1 compared to UT. Interestingly, the ability of TRX-m1 to promote GSAAT activity was lower than that of TRX-f1, as suggested above (**Figure 2D**). Like GSAATs from barley, *Sulfolobus solfataricus* and *Synechococcus* PC6803, the activity of GSAAT_At_ was also inhibited by the inhibitor gabaculine (Smith et al., 1991; Palmieri et al., 1996; Berry-Lowe et al., 1992). Lastly, incubation of GSAAT_At_ at 95°C for 10 min denatured and completely inactivated the protein (**Figure 2D**).

**Figure 3:**
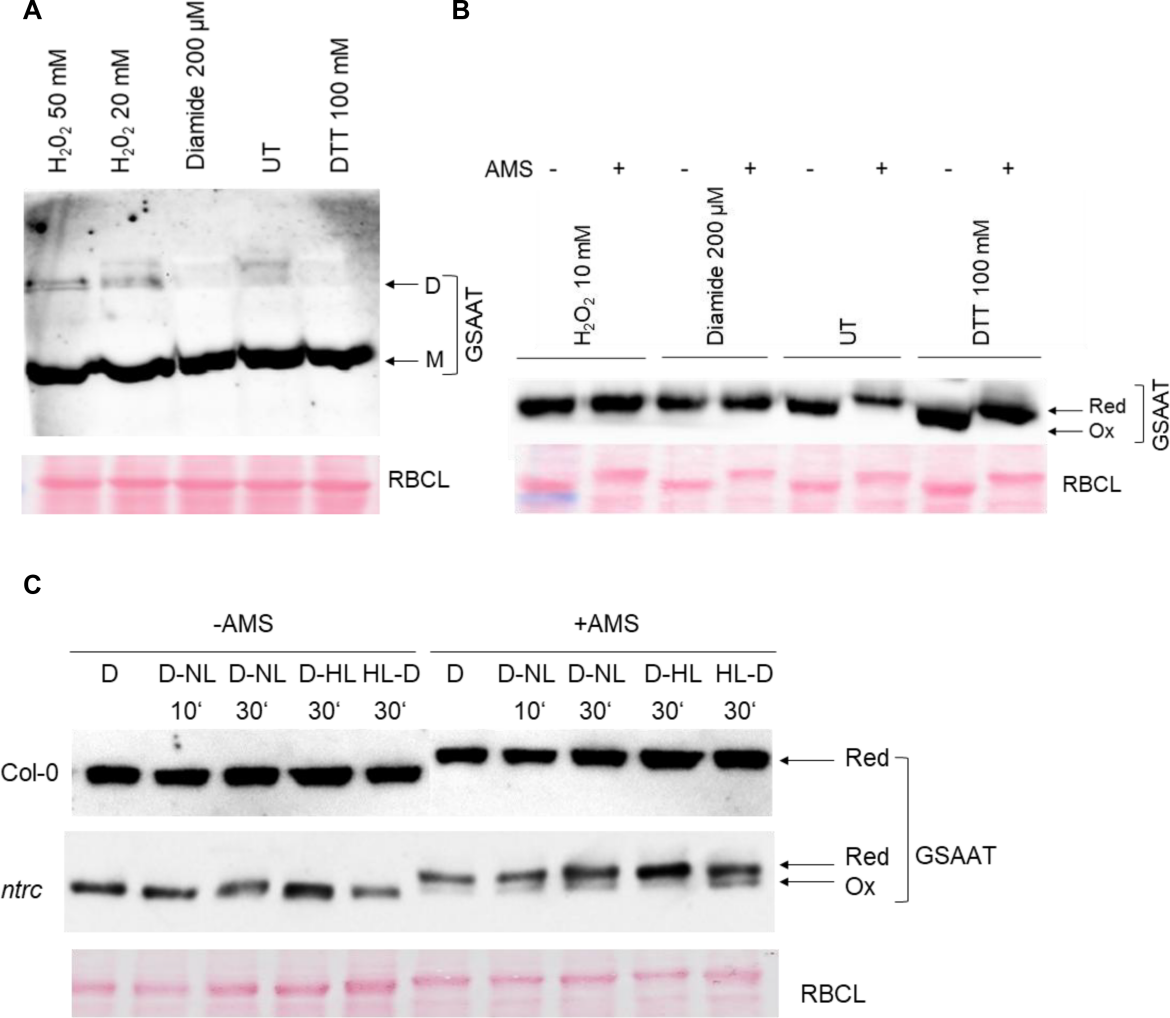
Analysis of the *in-vivo* redox state of Arabidopsis GSAAT. Leaf samples from wild-type and ntrc mutants were harvested at different time points (10 and 30 minutes) and light conditions (D, dark; NL, normal light: 120 μmol photons m^−2^ s^−1^; HL, high light-500 μmol photons m^−2^ s^−1^). **(A)** Wild-type extracts obtained from 3-week-old seedlings grown under short-day condition were pretreated with oxidizing agents (H_2_O_2_, diamide) and DTT, or left untreated (UT), for 30 min at room temperature (RT), and then fractionated on a non-reducing 8% SDS-PA gel (M, monomer; D, dimer). **(B)** Wild-type extracts were pretreated with oxidizing agents (H_2_O_2_, diamide), DTT or UT for 30 min at RT. After acid precipitation, proteins were incubated with 1 mM AMS for 30 min at RT. The proteins with or without AMS were separated on a 10% non-reducing SDS-PA gel. **(C)** Leaf samples from wild-type and *ntrc* mutants were harvested at different time points (after 10 and 30 min) and under different lighting conditions (D, dark; NL, normal light; HL, high light) and treated with 1 mM AMS. The proteins, together with the untreated (UT) extracts were separated on a 10% non-reducing SDS-PA gel. The arrows indicate the various redox states of the GSAAT monomer (red, ox) in **(B, C)**. Immunodetection was carried out using a GSAAT-specific antibody in **(A–C)**. Interestingly, the RBCL used as a loading also reacts with AMS, and therefore shows a mobility shift compared to the untreated protein samples **(B, C)**.

In addition to the *in-vitro* analysis of recombinant GSAAT_At_, the redox state of GSAAT in Arabidopsis leaf extracts was examined. Untreated (UT) and 30 min H_2_O_2_-treated leaf extracts from light-exposed wild-type seedlings contain some dimeric GSAAT, but the monomer is the dominant form. As diamide is a mild oxidizing agent compared to H_2_O_2,_ less dimers were observed in this case, as shown by electrophoresis on a non-denaturing PA gel (**Figure 3A**). Another experimental approach was undertaken to determine the relative proportions of the reduced and oxidized forms of GSAAT in Arabidopsis leaf extracts. The total protein extract was pretreated with oxidizing agents (H_2_O_2_, diamide) or with DTT for 30 min. Then, the free Cys residues of GSAAT_At_ molecules in the leaf extract were labeled with the sulfhydryl-binding reagent 1mM 4-acetoamido-4-maleimidylstilbene-2,2-disulfonic acid (AMS), and subsequently separated by non-reducing denaturing sodium dodecyl sulfate–PA gel electrophoresis (SDS-PAGE). AMS binds to reduced thiol groups in Cys residues and increases the molecular mass of the protein, so that the reduced and oxidized forms of GSAAT are readily distinguishable on SDS-PA gels, since the labeled protein (reduced form) migrates more slowly than the unlabeled oxidized form. In wild-type Arabidopsis leaf extracts grown under standard short day (SD) conditions, only one distinctive immunoreactive GSAAT band – either AMS-treated or UT – was observed. In samples treated with 10 mM H_2_O_2_ or 200 µM diamide, the GSAAT proteins were completely oxidized and therefore exhibited a mobility like that of the UT control. In contrast to the oxidized variants, the UT and DTT-treated samples contained reduced, slowly migrating GSAAT (**Figure 3B**).

We also evaluated the redox state of GSAAT in light- and dark-incubated wild-type and *ntrc* (SALK_012208; Serrato et al., 2004) seedlings during photoperiodic growth. These experiments revealed that after transfer from dark to either high light (HL; 500 μmol photons m−2 s−1) or normal light (NL, 120 μmol photons m−2 s−1) and vice versa, for either 10 min or 30 min, *ntrc* extracts contained two immunoreactive GSAAT bands – a partially oxidized species and a dominant reduced form – while wild-type leaves contained only the reduced from of GSAAT, regardless of whether dark-exposed leaf samples were analyzed after a D to NL (D-NL) or a D to HL (D-HL) transition, or light-exposed samples had undergone a HL-D transition (**Figure 3C**). These results conclusively demonstrate that, in the absence of NTRC, a portion of GSAAT_At_ remains in the oxidized form. In both gel blots shown in **Figures 3B, C**, RbcL was used as a loading control, and it too reacts with AMS, as indicated by a mobility shift relative to the UT protein samples.

### 3.3 Post-translational stability of TBS enzymes in TRX- and NTRC-deficient Arabidopsis seedlings

**Figure 4:**
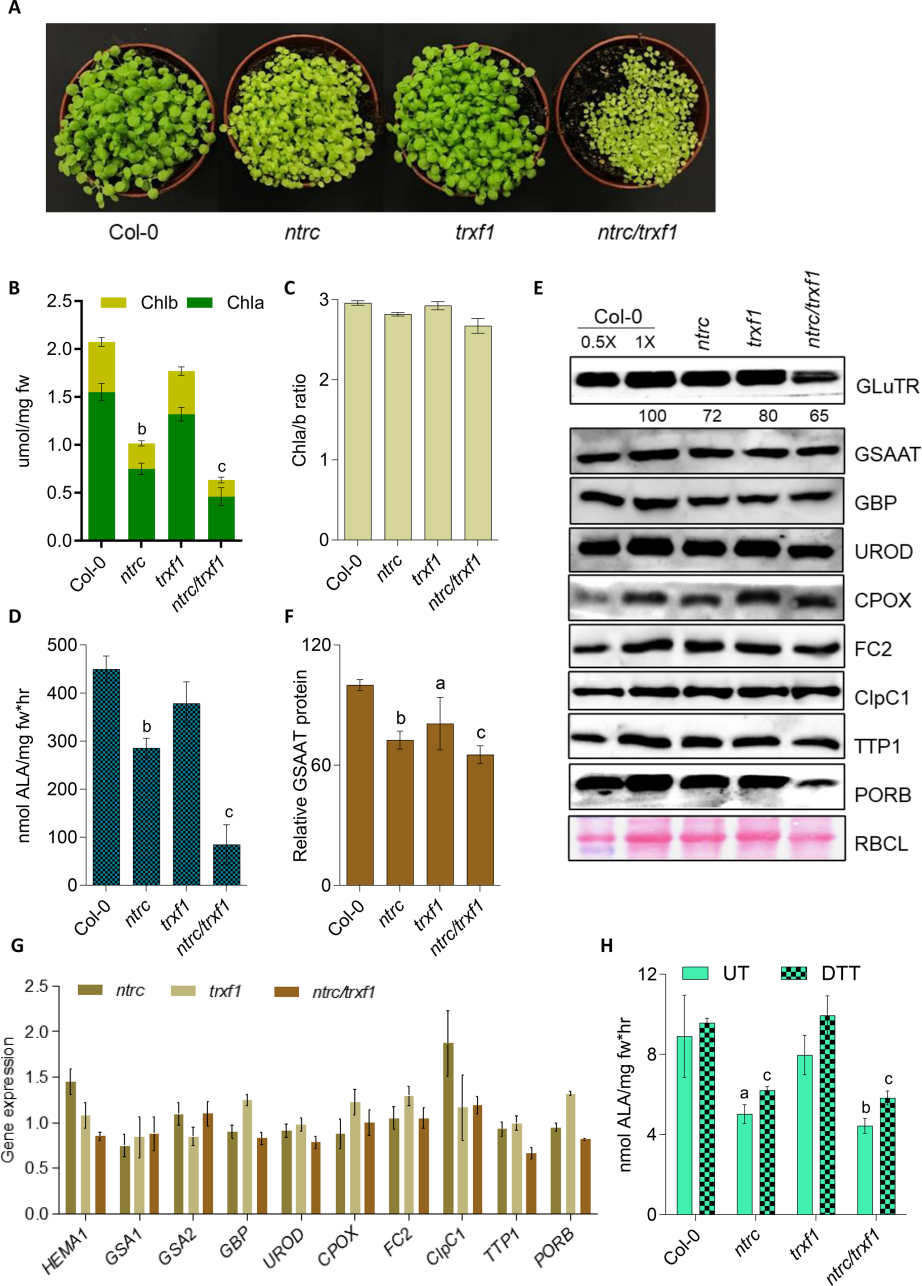
Analysis of Col-0, *ntrc*, *trxf1* and *ntrc/trxf1* mutants. **(A)** Three-week-old wild-type, and *ntrc*, *trxf1* and *ntrc/trxf1* mutant seedlings, grown under short-day conditions (10h/14h light/dark, 120 μmol photons m^−2^ s^−1^). **(B)** Comparison of their Chl a and Chl b contents. **(C)** Chl a/b ratios. **(D)** ALA-synthesizing capacity of detached leaves from 4-week-old seedlings. ALA, 5-aminolevulinic acid. Data in **(B), (C, D)** are presented as means of the standard deviation of three biological replicates each. Statistical significance compared with Col-0 seedlings is indicated by (a) *P* ≤0.05, **(b)***P*<0.01, **(c)***P*<0.001 based on Student’s t-test; fw, fresh weight; mg, milligram; hr, hours. **(E)** Levels of several TBS enzymes found in 3-week-old wild-type seedlings (Col-0), and mutant *trxf1, ntrc* and *ntrc/trxf1* seedlings grown under short-day conditions as revealed by immunoblot analysis. The Ponceau-stained large subunit of RuBisCO (RBCL) served as a loading control. GluTR, glutamyl-tRNA reductase; GSAAT, glutamate 1-semialdehyde aminotransferase; UROD, uroporphyrinogen III decarboxylase; CPOX, coproporphyrinogen oxidase; FC2, ferrochelatase 2; ClpC1, caseinolytic protease 1; TTP1, TBS-regulating tetratricopeptide repeat protein 1. The numbers in the immunoblot represent the normalized abundancies of GSAAT in the analyzed *ntrc*, *trxf1* and *ntrc/trxf1* mutants relative to the Col-0 seedlings using three western blot replicates. **(F)** Quantitative analysis of GSAAT proteins shown in the immunoblot. **(G)** Relative expression levels of various TBS transcripts in leaves of 3-week-old wild-type seedlings and the three mutant reducing enzymes. *HEMA1*, encoding glutamyl-tRNA reductase. **(H)** The GSAAT activity of the soluble protein fraction was measured from leaf extracts of three-week-old Col-0, *ntrc, trxf1 and ntrc/trxf1* seedlings grown under short-day conditions. The assay was performed with and without (UT) 1 mM DTT. The data in **(F–H)** indicate the means and SD of three biological replicates. Statistical significance of the differences between the mutants relative to Col-0 plants is shown by a, *P*≤ 0.05, b, *P*≤ 0.01, c, *P*≤ 0.001 based on Student’s t test. Data in **(E)** correspond to the means and standard deviations (SD) of three independent western blots.

We also addressed the redox sensitivity of GSSAT *in planta* using three-week-old seedlings of *ntrc, trxf1* (SALK_128365; Thormählen et al., 2015)*, ntrc/trxf1* and wild type grown under SD conditions under normal lighting (NL, 120 µmol photons m^-2^ sec^-1^, **Figure 4A**) to assay for GSAAT accumulation and activity. Knockout of the *NTRC* gene in Arabidopsis resulted in a growth-retarded, pale green mutant phenotype with 50% less Chl than in the wild type (**Figure 4B**). With 14% less Chl, the *trxf1* mutant is phenotypically indistinguishable from the wild type, while *ntrc/trxf1* exhibited an additive effect with severe growth retardation and 69% less Chl compared to wild type (Thormählen et al., 2015; and **Figures 4A, B**). Despite the decrease in Chl levels, the Chl a/b ratio did not change in either mutant or WT seedlings (**Figure 4C**). The ALA-synthesizing capacities of *ntrc, trxf1* and *ntrc/trxf1* mutants were diminished by 36%, 15% and 80% compared to WT seedlings, respectively (**Figure 4D**), thus confirming that the absence of NTRC is responsible for impaired ALA synthesis and decreased Chl content in single and double mutants (Richter et al., 2013; 2018; Wittmann et al., 2018; 2024).

We then analyzed the stability and activity of GSAAT in order to verify the contribution of redox-dependent control to the regulation of ALA synthesis. Immunoblots of the leaf extracts confirmed a lower content of GSAAT and some other TBS proteins (such as PORB, GluTR and uroporphyrinogen decarboxylase (UROD)) in the *ntrc/trxf-1* double mutant relative to wild-type seedlings (**Figures 4E, F**), and also revealed a slight decrease in accumulation of GSAAT, as previously reported (Wittmann et al., 2018). The relative GSAAT protein content fell by 22%, 12% and 35% in *ntrc, trxf1* and *ntrc/trxf*1, respectively, compared to WT, as quantified in three different immunoblot experiments (**Figure 4F**). These decreases in GSAAT content cannot be explained by reduced transcriptional activity of the corresponding *GSA1* and *GSA2* genes (**Figure 4G**). This observation is compatible with the lack of correlation between constant transcript content and lower levels of GluTR, UROD and PORB (**Figures 4E, G**). The GSAAT activity in leaf extracts decreased by 30%, 10% and 40% in *ntrc, trxf1* and *ntrc/trxf1*, respectively, indicating a correlation between the decreases in plastidal reductants, GSAAT level and GSAAT enzymatic activity (**Figures 4E, F, H**). Pre-incubation of the leaf extracts with 2 mM DTT led to moderate increases of about 23%, 20%, and 30% in GSAAT activity in *ntrc, trxf1, and ntrc/trxf1*, respectively, compared to the UT extracts (**Figure 4H**). We assume that most of the GSAAT in all plant variants is present in the reduced form. Hence, the significant decrease in the rate of ALA synthesis in *ntrc* and *ntrc/trxf1* mutants can be explained by decreased amounts of GluTR and GSAAT as the result of a relative lack of reductants.

### 3.4 GSAAT interacts with TRX isoforms and with NTRC

**Figure 5:**
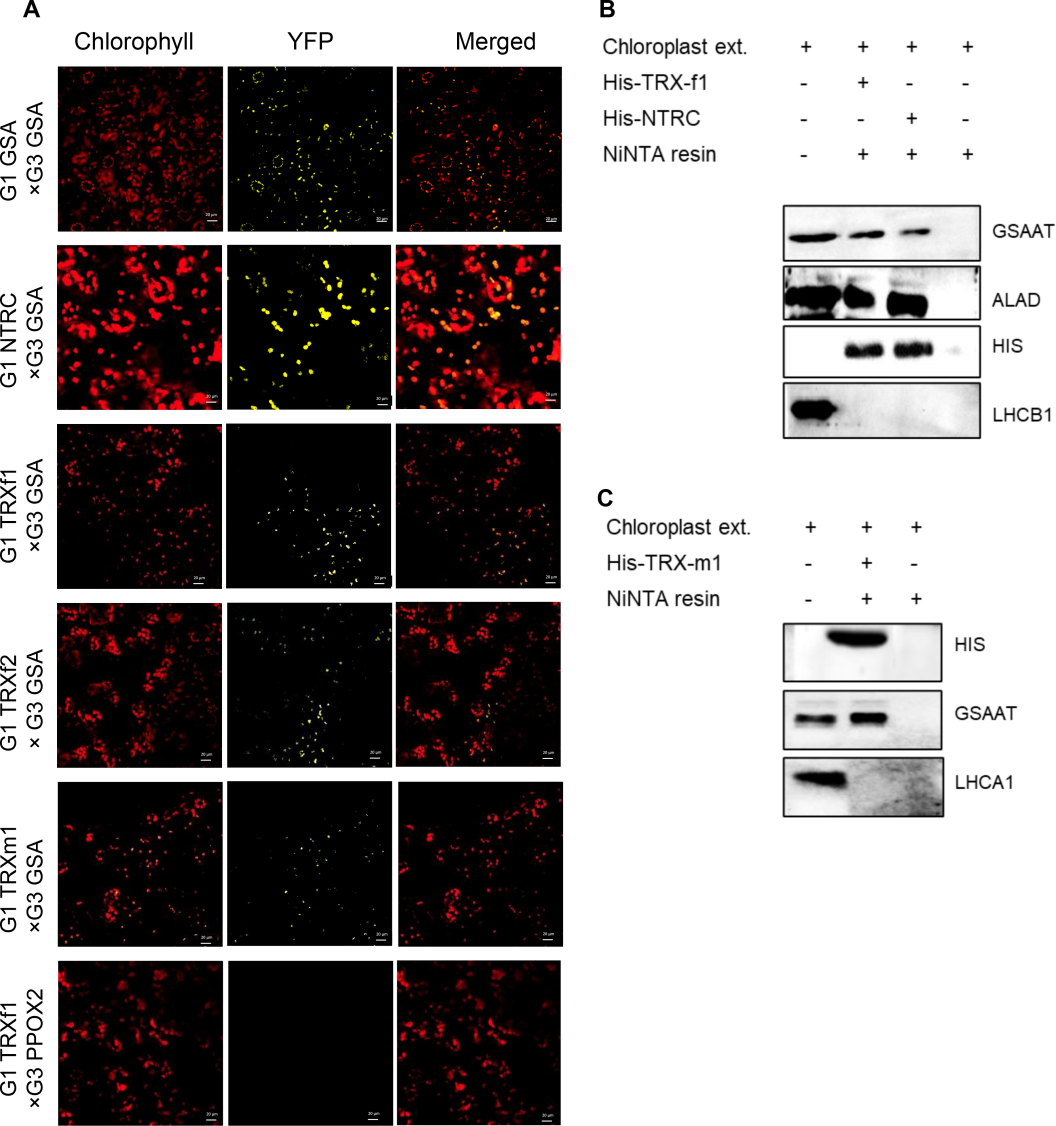
Physical interactions between GSAAT and NTRC or TRXs. The interaction between GSAAT and the various redox regulators was demonstrated by **(A)** bimolecular complementation (BiFC) and **(B)** pull-down assays. **(A)** Images show *Nicotiana benthamiana* leaves, following infiltration with *Agrobacterium tumefaciens* strains expressing different halves of either GSAAT or TRXs and NTRC fused to G1 (encoding N-terminal YFP, pVyNE) and G3 (encoding C-terminal YFP, pVyCE) plasmids, visualized under the confocal microscope. Left column: autofluorescence of chlorophyll; middle column: YFP; right column: merged images. A combination of PPOX2 and TRX-f1 was used as the negative control. Scale bars in all figure panels correspond always to 20µm. **(B)** The recombinant, His-tagged bait proteins TRX-f1 and NTRC were incubated with chloroplast extracts. Proteins in the eluate were detected by immunoanalysis using antibodies against GSAAT, ALA-dehydratase (ALAD), His-tag and Lhcb1. The ALAD protein served as the positive control, while the Lhcb1 protein was used as the negative control. **(C)** The recombinant, His-tagged bait protein TRX-m1 was incubated with chloroplast extracts. GSAAT in the eluate was immunologically detected by using the anti-GSAAT antibody. The Lhcb1 was used as the negative control.

We used different methodological approaches to confirm the interactions of GSAAT with TRXs and NTRC. First, a bimolecular fluorescence complementation (BiFC) assay was performed. Gene constructs encoding fusion proteins consisting of either the C- or N-terminal half of the yellow fluorescent protein (YFP) and the proteins of interest were transiently expressed together in leaves of Nicotiana benthamiana after infiltration with Agrobacterium strains. After two days of incubation in the dark, the YFP signal was observed by confocal laser-scanning microscopy (**Figure 5A**). The results confirm the interaction of GSAAT with the plastidal reductants NTRC, TRX-f1, TRX-f2 and TRX-m1. Parallel expression of GSAAT variants bearing either the N- or the C-terminal half of YFP and fusion constructs of *TRXf1* and *protoporphyrinogen oxidase 1* (*PPOX1)* were used as positive and negative controls, respectively.

Secondly, an *in vitro* pull-down experiment was performed with recombinant His-tagged TRX-f1, TRX-m1 and NTRC as bait proteins, in order to trap potential target proteins in the chloroplast extracts. The ALAD protein, which is a known target for TRXs and NTRC, was used as a positive control (Wittmann et al., 2024). GSAAT was found in the elution buffer after release from the specifically bound reductants in both experiments (**Figures 5B, C**). As NTRC, TRX-f1 and TRX-m1 were tagged with 6X-His tag, the immune reactions with the anti-His antibody were observed in all three His-tagged proteins, which were bound to the Ni-NTA resin. The light-harvesting chlorophyll a/b binding protein 1 of photosystem II (LHCB1) and photosystem I LHCA1 did not interact with NTRC, TRX-f1 or TRX-m1, respectively, in these assays.

In conclusion, these results indicate potential interaction of GSAAT with the plastidal reductants NTRC, TRX-f1 and TRX-m1. As it is obvious that TRX-interaction with its target proteins occurs only transient, we do not speculate on a tight binding. However, this finding is consistent with the outcomes of thiol-dependent affinity chromatography of TRX-f1 and TRX-m1, in which GSAAT was pulled down from extracts of *Spinacia oleracea* (Balmer et al., 2003), and with analyses of the Chlamydomonas thioredoxome (Perez-Perez et al., 2017).

### 3.5 *In-vitro* modifications of the structure and activity of GSAAT cysteine substitution mutants under redox conditions

To examine the relevance of the conserved Cys residues of GSAAT_At_ for its structure and activity, the WT and the four single Cys (C) to Ser (S) substitution mutant proteins - designated as GSAAT(WT), GSAAT(C138S), GSAAT(C168S), GSAAT(C190S) and GSAAT(C396S), respectively - and the double mutant GSAAT(C168S/C190S), were heterologously expressed as His-tagged proteins and subsequently purified. Although the majority of all recombinant GSAAT_At_ mutant variants were insoluble after induction of their expression in *E. coli*, a significant fraction of each of the (approximately 50 kDa) heterologous proteins remained soluble and were analyzed by non-reducing SDS-PAGE for redox-dependent switching between monomeric and dimeric states with CuCl_2_ as an oxidizing agent and DTT as the reducing agent.

**Figure 6:**
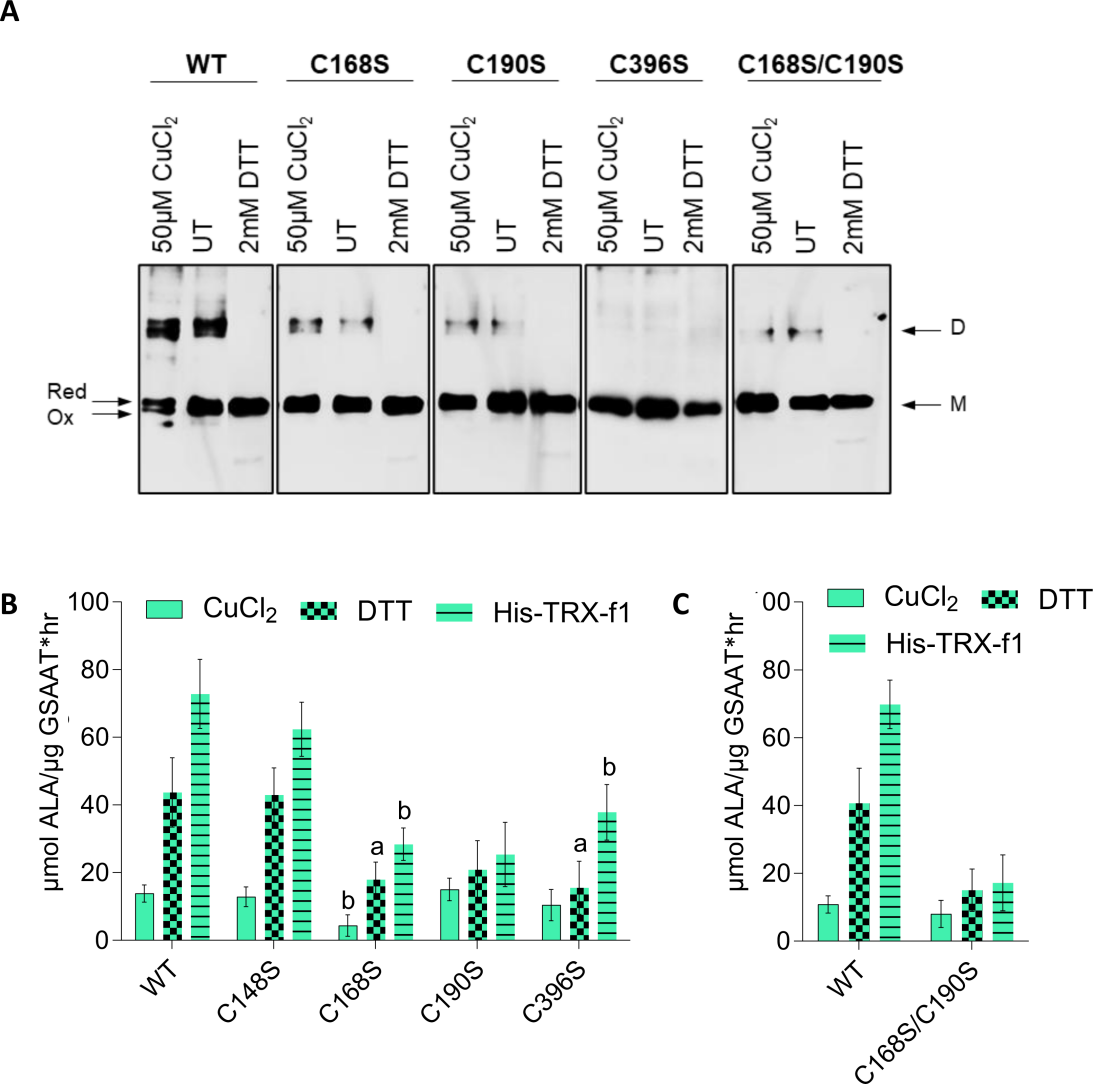
**(A)** Redox-dependent structural modifications in recombinant GSAAT. The GSAAT(WT) and three single cysteine **(C)** to serine (S) substitution mutants [GSAAT(C168S), GSAAT(C190S), GSAAT(C396S), and the double mutant GSAAT(C168S/C190S)] were heterologously expressed as His-tagged proteins and subsequently purified. Later, 500-ng aliquots of each of these proteins were either pre-incubated with an oxidant (Ox, 50 µM CuCl_2_) or reducing agent (Red, 2 mM DTT) for 15 min at room temperature. The samples were then fractionated by non-reducing SDS-PAGE (10% gel). The bands were detected after Western blot transfer using a His-tag-specific antibody. **(B)** Effects of redox-dependent modifications of recombinant GSAAT. GSAAT(WT) and the four single Cys **(C)** to Ser (S) substitution mutant proteins C138S, C168S, C190S, C396S, and the double mutant C168S/C190S were either left untreated (UT), or pre-incubated with an oxidant (50 µM CuCl_2_) or reducing agents (2 mM DTT or 3 µM HisTRX-f1) for 15 min at room temperature prior to the 10 min enzyme assay as described in chapter 2.6. Amounts of ALA formed were photometrically measured using Ehrlich’s reagent following the method of Mauzerall and Granick (1956). The data indicate the means and standard deviations of three replicates each. Statistical significance of the mutants compared to GSAAT(WT) is shown by a, *P*-value≤ 0.05, b, *P*-value<0.01, c, *P* ≤ 0.001 based on Student’s t-test.

We verified dimer formation and *in-vitro* activity of purified recombinant wild-type and mutant GSAAT_At_. GSAAT dimerization was examined in UT protein samples, and after the addition of 50 µM CuCl_2_ or 2 mM DTT. All of the GSAAT variants were separated under reducing conditions as the 50-kDa monomer. Under oxidizing conditions, two monomeric redox states (red, ox1) could be distinguished, as the monomer migrates as a double band in GSAAT(WT) proteins and points to an independent intra-molecular redox modification. GSAAT(C396S) accumulated only in a monomeric form, while the other mutant variants were found in almost similar amounts of monomers and very small amounts of dimers in the respective UT sample and under oxidized conditions (**Figure 6A**). GSAAT(C138S) mimicked the structural properties and the mobility of GSAAT(WT) (not shown), therefore, a participation of Cys138 in the formation of redox dependent intra- and inter-molecular disulfide bridges are excluded. In summary, with the exception of GSAAT(C396S), fractions of GSAAT(WT) and, to a lesser extent, the other GSAAT mutants accumulate as 100 kDa dimers under non-reducing conditions.

We then examined the catalytic activity of the recombinant GSAAT_At_ substitution mutants under different redox conditions. GSAAT(WT), the four single and the double C→S mutants were initially oxidized in 50 µM CuCl_2_ and then directly reduced by the addition of either 2 mM DTT or 3 µM recombinant His-TRX-f1. The oxidized forms of the six variants displayed similarly low GSAAT activities. Maximal GSAAT activity (relative to DTT treatment) was observed upon addition of TRX-f1. GSAAT(WT) and GSAAT(C138S) showed the biggest increases in activity – 215% and 238% and 426% and 384% in the presence of DTT and TRX-f1, respectively. This analysis also indicates that the purified GSAAT was not completely reduced. The reduced GSAAT(C168S), GSAAT(C190S) and GSAAT(C396S) mutants, as well as the double mutant GSAAT(C168S/C190S), each exhibited only half as much activity as that measured in the presence of DTT or TRX-f1 (**Figures 6B, C**). In fact, whether incubated with DTT or TRX-f1, the residual activity of the oxidized proteins GSAAT(C190S) and GSAAT(C168S/C190S) could scarcely be enhanced at all, which points to an impaired redox sensitivity of these mutants. We therefore assume that these mutant variants neither respond to CuCl_2_ inhibition nor to DTT/TRX-f1 stimulation, and instead behave as redox-unresponsive proteins. However, the GSAAT(C396S) mutant showed a moderate stimulation of its activity (by 48% and 262% relative to the oxidized protein), when treated with DTT and TRX-f1, respectively. In light of their redox insensitivity, we suggest that GSAAT(C168S) and GSAAT(C190S) are the most likely candidates for the formation of an intramolecular disulfide bridge, while the Cys residues 138 and 396 apparently do not to contribute to the redox-sensitive activation of GSAAT.

### 3.6 Expression of *GSA1* genes bearing Cys→Ser substitution mutants in the *gsa2* mutant background

We then constructed GSAAT_At_ mutants in which the conserved Cys residues had been individually replaced by Ser, and assessed their stability, activity and redox states in a *gsa2* background. Previous studies revealed GSAAT2 as the dominant isoform in Arabidopsis leaf extracts, and *gsa2* showed a stronger pale green phenotype than *gsa1* (Sinha et al., 2022). To this end, a homozygous *gsa2* strain (GK_362C09) was transformed with *p35S:GSA1* mutant gene constructs by *Agrobacterium*-mediated transformation. The *gsa2* strain itself is known to show a stronger GSAAT deficiency phenotype than its *gsa1* counterpart (Sinha et al., 2022). We then selected single transgenic lines that expressed comparable levels of each of the GSAAT1 variants in the homozygous *gsa2* background (**Supplementary Figure 3A**). All selected transgenic lines were morphologically wild-type-like. Moreover, none of the homozygous *gsa2* mutants expressing any of the different transgenic *GSA* variants showed any visible phenotypic anomaly (**Supplementary Figure 3A**). Chl content and ALA-synthesizing capacity consistently remained wild-type-like (**Supplementary Figures 3B, C**) – with the striking exception of GSAAT(C190S), which exhibited a significantly lower rate of ALA synthesis. Owing to use of the 35S promoter for the expression of the *GSAAT_At_* variants, GSAAT1 was overproduced in all the transgenic lines. However, GSAAT(C190S) accumulated to a lesser extent than any of the other mutants, albeit still more than that expressed in the *gsa2* mutant. All other TBS proteins analyzed were expressed at similar levels in all transgenic lines (**Supplementary Figure 3D**). Because of the overexpression of the transgene, the complementation efficiency of the C→S substitution variants of GSAAT1 could not be assessed. Comparisons between the transgenic GSAAT1 substitution mutants and wild-type GSAAT1 expressed in the *gsa2* background revealed a slightly reduced ALA synthesis capacity of leaf discs and *in planta* GSAAT activity of the leaf extracts only for the GSAAT(C190S) line (**Supplementary Figures 3C, E**). The slight decrease in the accumulation of GSAAT(C190S) is probably due to impaired redox-dependent protein stability or enzyme activity.

We also assessed the *in-planta* effects of altered redox conditions on the enzyme activities of the wild-type GSAAT1 and its substitution mutants, and analyzed GSAAT1 activity of chloroplasts in the presence or absence of DTT and CuCl_2_. Under oxidizing conditions, a drastic decrease in GSAAT activity in leaf extracts is observed for all transgenic lines, which is similar to that seen with GSAAT(WT). UT extracts of all transgenic lines exhibited at least two- to three-fold higher GSAAT activity, indicating that GSAAT was mainly present in reduced form. Interestingly, GSAAT(WT), GSAAT(C138S) and GSAAT(C396S) lines were still markedly redox responsive, and exhibited up to 20% higher enzyme activity when supplied with DTT relative to the UT control conditions, while the two *gsa2* lines expressing GSAAT(C168S) and GSAAT(C190S) resulted only in 9% and 2% increased GSAAT activity upon addition of DTT to the extracts (**Supplementary Figure 3E**). Hence, mutation of either C168 or C190 results in the loss of the redox responsiveness of GSAAT1 *in planta*.

In addition, the electrophoretic mobility of GSAAT(WT) and the GSAAT(C→S) lines is altered by the thiol-reactive compound AMS. As proposed as result of the *in planta* enzyme activities, this finding confirms that most of the GSAAT protein is always predominantly reduced in planta under standard light conditions (**Supplementary Figure 3F**).

### 3.7 Structural insights into the mode of action of the thiol switch in GSAAT1

**Figure 7:**
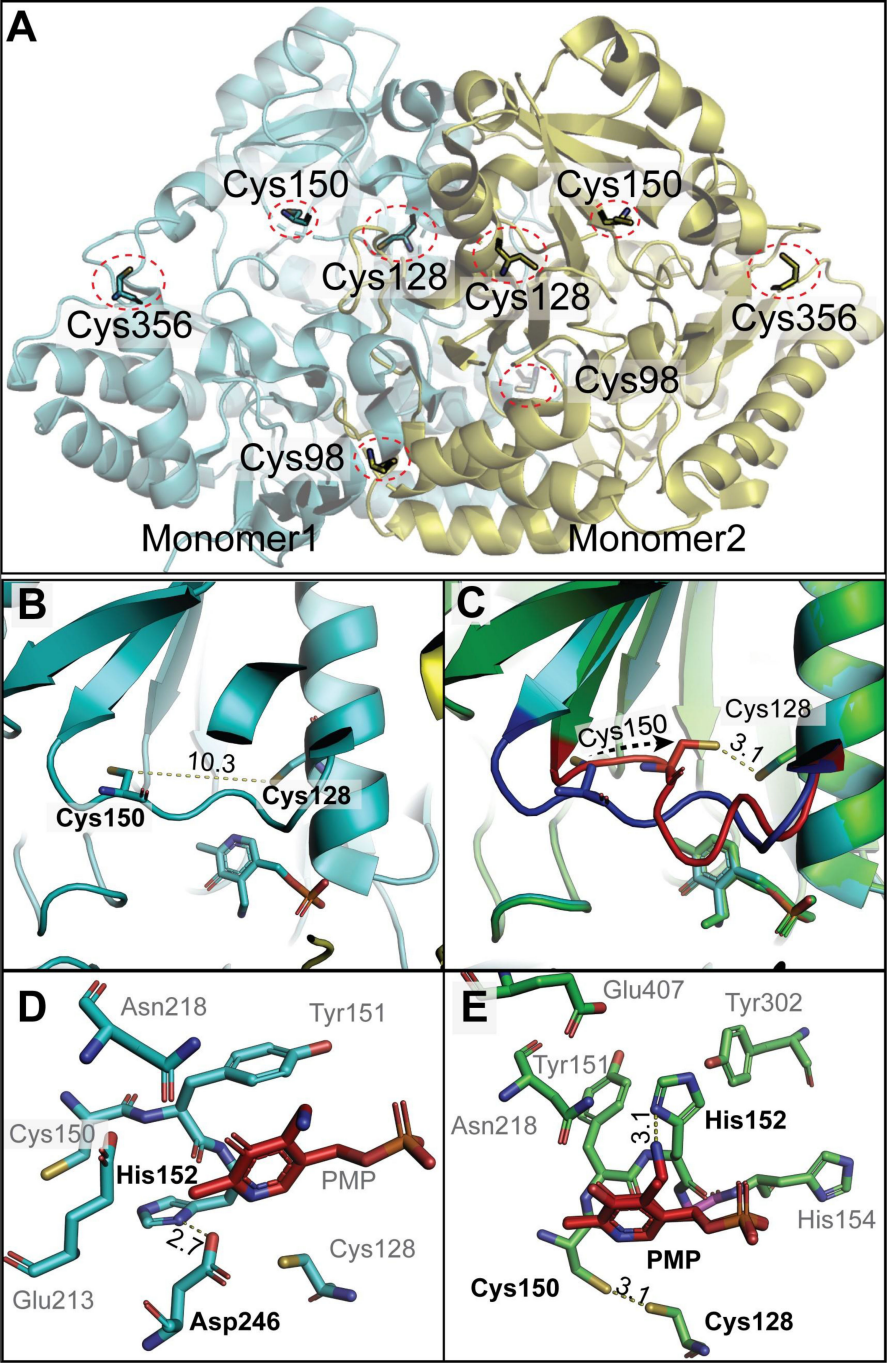
Structural insights into dimeric GSAAT from Arabidopsis (PDB ID: 5hdm; Song et al., 2016). **(A)** The dimeric GSAAT1 structure shows the positions of conserved Cys residues at positions 138 (98 in the model), 168 (128 in the model), 190(150 in the model), and 396 (356 in the model), each marked by red circles. **(B)** Zoomed-in view detailing the distance between the sulfur atoms of Cys128 and Cys150. **(C)** A modified model by COOT emphasizes the movements of the flexible loop, leading to the proximity of Cys128 and Cys150. The Blue loop is the actual position of the residue in the detected structure; however, the red one is the tested model. **(D)** The local environment around the PMP within the GSAAT protein illustrates potential hydrogen bonds between His152 and Asp246 (corresponding to His 192 and Asp 286 in the text). **(E)** Alterations in the local environment around the PMP in the COOT modified model, suggesting changes that may impact PMP interaction and enzyme function. All distances shown are in angstrom. The structures are visualized using PyMol (www.pymol.org).

The X-ray structure of the Arabidopsis GSAAT1 dimer has been reported at 1.25 Å resolution (Song et al., 2016). The structural model of GSAAT_At_ (PDB ID: 5hdm) revealed that Cys138, Cys168 and Cys190 are more buried within the dimeric structure, while Cys396 is exposed on the surface of GSAAT, albeit mirror symmetrically on opposite sides of the dimer (**Figure 7A**). This conformation would exclude intermolecular disulfide bonding for dimerization, as suggested above. The two residues Cys168 and Cys190, which are proposed to be highly sensitive to redox changes, show on one hand the closest proximity to each other, although the distance between the sulfur atoms of both Cys residues is still around 10.3Å. On the other hand, they are certainly more accessible for TRX in the monomeric form than in the dimeric form. But we still propose that TRX may have access to the side groups of Cys190 and likely Cys168, (**Figure 7B**). We speculate that conformational movement causing a structural rearrangement of the flexible loop may bring these two Cys residues closer. Testing modeling positions of the amino acid residues in the flexible loop without resulting in any steric clash using Coot (Emsley et al., 2010), allowing Cys190, situated in the flexible loop, to approach Cys168 without hindrance. The resultant distance between the sulfur atoms of the two Cys residues could decrease to less than 3Å (**Figure 7C**). The distance could even be further reduced to ≈2.2 Å through a slight movement and rotation of Cys168, which would permit the formation of an intra-molecular disulfide bond. Such a re-arrangement event has also been suggested for the disulfide bonding of oxidized Mg protoporphyrin methyltransferase (CHLM), another redox-controlled enzyme of Chl biosynthesis (Richter et al., 2017).

## 4 Discussion

The primary goal of this investigation was to elucidate the role of the redox-sensitive Cys residues in Arabidopsis GSAAT1 (GSAAT_At_) by investigating the stability and catalytic activity of its Cys→Ser substitution mutants. Several criteria characterize GSAAT1 as a redox-sensitive protein. Dimerization of purified recombinant GSAAT is favored under oxidizing conditions, while reducing agents lead to formation of its monomers (**Figures 2A, B**). In addition to the *in-vitro* examination of recombinant GSAAT1, the oxidized state of the enzyme in Arabidopsis extracts also resulted in a partial accumulation of a dimeric fraction of GSAAT (**Figure 3A**). Thus, AMS-treated GSAAT shows the distinct electrophoretic mobility shifts of a reduced and an oxidized form of GSAAT (**Figure 3B**). Moreover, NTRC-deficient Arabidopsis plants also exhibited two immunoreactive monomeric GSAAT bands after oxidation treatment, while the wild-type plant extract contained entirely reduced GSAAT (**Figure 3C**). Both the NEM-treated oxidized and UT recombinant GSAAT_At_ enzymes also showed two additional oxidized GSAAT variants (**Figure 2C**). This suggests the potential of two oxidizable thiol groups among the enzyme’s Cys residues.

Relative to the UT and oxidized protein, the GSAAT activity was always enhanced under reducing conditions (**Figure 2D**). In addition, the GSAAT1 is more susceptible to the reducing activity of TRX-f1 than to that of TRX-m1 (**Supplementary Figure 2**). *In planta*, wild-type GSAAT seems to be entirely reduced in both the light and the dark (**Figure 3C**). In *ntrc* seedlings, two GSAAT bands indicate the presence of a partially oxidized and a dominant reduced form during transitions from darkness to high or normal light (HL and NL), and *vice versa*; in wild-type seedlings, the reduced form was always visible. This implies that a portion of GSAAT is retained in an oxidized state in the absence of NTRC, as indicated by its increased mobility and a possibly more compact, globular protein structure (**Figure 3C**).

The diminished accumulation of GSAAT attributable to TRX-f1 and NTRC deficiency results in a decrease in the flow of metabolites through the TBS pathway, and a correspondingly lower Chl content (**Figures 4A, B, E, F**), but does not alter the Chl a/b ratio (**Figure 4C**). Given the sequence similarity and identity of the Cys residues of both GSAAT isoforms, it can be assumed that the redox properties of GSAAT1 could also apply to GSAAT2.The *ntrc/trxf-1* seedlings showed significantly reduced GSAAT stability, which is reminiscent of the accumulation of other redox-controlled TBS proteins, such as ALAD, CHLI, and CHLM (Ikegami et al., 2007; Luo et al., 2012; Richter et al., 2013; 2018; Wittmann et al., 2018; 2024). The stability of GSAAT *in planta* is more markedly compromised by deficiency of NTRC or TRX-f than is its catalytic activity (**Figure 4H**). It should be mentioned that lack of reductants and diminution of reducing power is primarily correlated with lower TBS protein content (Wittmann et al., 2024). This redox-dependent control mechanism differs from the redox-dependency of other plastid-localized enzymes, such as those involved in the Calvin-Benson cycle or starch metabolism. It appears that oxidized enzymes of the Calvin-Benson cycle are more likely to be inactivated, while oxidized TBS enzymes seem to be more destabilized (Buchanan, 2016; McFarlane et al., 2019; Michelet et al., 2013; Eliyahu et al., 2015; Wittmann et al., 2023).

### 4.1 Identification of redox-sensitive cysteine residues of GSAAT_At_

To identify the redox-sensitive Cys residues in GSAAT, we examined how the redox-dependent control of GSAAT affects its stability and catalytic activity. Mutant transgenes bearing single Cys→Ser substitutions in GSAAT_At_ were generated and subcloned for overproduction of the recombinant proteins in *E. coli* and for expression of the GSAAT1 mutants in Arabidopsis *gsa2*. The *gsa2* mutant was selected because its seedlings show fainter green leaves than *gsa1* (Sinha et al., 2022), which allows the efficiency of the GSAAT1 substitution mutants to be clearly demonstrated in terms of their ability to complement the deficiency of the dominant GSAAT2 enzyme.

When the soluble recombinant GSAAT_At_ variants were electrophoretically separated on a non-reducing PA gel, GSAAT(WT) was found to contain almost equal amounts of the monomer and dimer forms (**Figure 6A**), while GSAAT(C168S) and GSAAT(C190S) expressed only small amounts of the dimer, and GSAAT(C396S) did not migrate as a dimer (**Figure 6A**). The monomerization effect after adding DTT or TRX is of course interesting, especially, as the monomeric interface does not show any disulfide bond (**Figure 7**) and the GSAAT(C396S) mutant in *E. coli* was found only as monomer. It remains not clear why this surface residue Cys396, located far away from the monomer interaction could lead to monomerization. On the other hand, the GSAAT(C396S) mutant shows increased activity in the presence of a reducing agent, further confirming the redox function mediated by other cysteines (**Figure 6**). Apart from the low propensity of the GSAAT(C168S) and GSAAT(C190S) mutants to dimerize, these single substitution mutants not only failed to promote redox-dependent dimerization and oxidation of monomeric GSAAT, but even prevented it. Hence, we propose that the oxidation state of the monomeric GSAAT depends on the redox status of several Cys residues. Moreover, it is hypothesized that Cys168 and Cys190 can form an intra-molecular disulfide bond in wild-type GSAAT.

Neither the dimer nor the additional monomeric form (Ox) were detectable for GSAAT(C396S). The complete absence of dimerization in C396S suggests that Cys396 may be involved in an inter-molecular disulfide bond that results in the formation of an oligomeric structure that combines GSAAT with other enzymes, factors or molecular chaperones involved in ALA formation, such as GluTR, GBP (Sinha et al., 2022) and tetrapyrrole biosynthesis-regulating tetratricopeptide-repeat protein1 (TTP1) (Herbst et al., 2023). It is likely that the formation of a putative inter-molecular disulfide bond depends on the interaction between a surface Cys on GSAAT and Cys residues in other proteins, facilitating the assembly of a bimolecular or oligomeric complex which may play a critical role in stabilizing the protein structure in a dimer form.

The purified UT recombinant GSAAT variants were not entirely reduced, and additional reducing power enhanced their enzyme activities. The GSAAT(C138S) mutant displayed a level of catalytic activity similar to that of the wild-type enzyme. The enzymatic activity of GSAAT(WT) increased by 3.2 and 5.3-fold upon addition of DTT and TRX-f1, respectively, relative to the UT and oxidized protein. Similarly, GSAAT(C138S) showed 3.3- and 4.9-fold elevated activities in the presence of reductants relative to the UT GSAAT mutant. Regarding the redox-dependent structural changes in the recombinant GSAAT proteins, the mobility of the GSAAT(C138S) mutant was found to be similar to the wild type under various conditions, suggesting that a role of Cys138 in the formation of redox-dependent intra- and intermolecular disulfide bridges can be excluded (data not shown). These observations suggest that GSAAT(C138S) mimics the catalytic and structural properties of GSAAT(WT) (**Figure 6B**).

Supplementation with TRX-f1 enhanced the enzymatic activity of GSAAT(C396S) by 3.6-fold. However, the activities of its UT and oxidized forms were 1.3-fold lower than that of the wild-type. In contrast, the other two substitution mutants, GSAAT(C168S) and GSAAT(C190S), and the corresponding double mutant, displayed hardly any increase in activity under reducing conditions. These observations from enzyme assays lead us to propose that the Cys residues at positions 168 and 190 are essential for the protein activity and are likely sensitive to redox changes.

In *gsa2* seedlings, the 35S promoter-driven expression of the Cys→S substitution mutants of GSSAT resulted in wild-type-like green pigmentation of the leaves, which indicates that the GSAAT2-deficient phenotype can be rescued by all GSAAT1 variants (**Supplementary Figure 3A**). This points to the capacity of all overproduced mutant GSAAT1 variants to compensate for the loss of GSAAT2. Assays performed with leaf extracts of these lines indicated that GSAAT(C138S) and GSAAT(C396) are weakly stimulated by the addition of DTT (by up to 20% compared to their UT samples). However, the GSAAT(C168S) and GSAAT(C190S) variants are hardly stimulated at all by additional reducing power. Moreover, GSAAT(C190S) also appears to be less stable than the other C→S substitution mutants (**Supplementary Figure 3D**).

### 4.2 Consequence of the predicted mode of action of the thiol switch in GSAAT1

Structural insights into GSAAT that led us to speculate about the mechanism of action of the thiol switch prompts other questions, which we would like to discuss. 1. How can this rearrangement of the loop be facilitated? The answer must take into account not only the formation of the GSAAT dimer, but also the recently proposed oligomeric structure of an ALA-synthesizing complex (Sinha et al., 2022). More specifically, one must consider that several factors could play a role in the conformational rearrangement, including changes in pH and redox states in the immediate environment. Other reasons could also promote this conformation change that facilitates the two Cys residues to come into close spatial proximity and facilitate the formation of disulfide bonds. Based on the published structure (Song et al., 2016), we suggest a possible hydrogen bonding between His192 and Asp286 with PMP due to their local proximity of a distance of 2.7 Å (**Figure 7D**). By sharing a hydrogen of His192 and Asp286, this possible hydrogen bond interaction could be part of a network of interactions that influences the conformation and dynamics of the protein. Thus, we propose that these structural rearrangements are due to the positioning of the hydrogen bond, which could affect the flexibility of nearby loops, and possibly trigger the movement of the Cys190-containing loop (**Figure 7D**).

2. How can the reduced activity of oxidized GSAAT be explained? How can disulfide bonding lead to reduced activity? Once the disulfide bridge is formed as a result of loop rearrangement, the bound PMP could potentially be accessible to additional interaction partners (**Figure 7E**). We suggest that the hydrogen rearrangement could take place between His192 in the flexible loop and Asp286. Both residues are within an ideal hydrogen bond distance from the N-atom of the ϵ-amino group of PMP after the formation of the Schiff-base linkage between K274 (K314 of the precursor protein) and PLP (the N-atom of the bound PMP/PLP). Such additional interaction, facilitated by the disulfide bridge, may altered the enzyme conformation, effecting its catalytic efficiency.

3. What consequences might a disulfide bond formation between Cys168 and Cys190 have for the action of the cofactor PMP? The structural change (**Figure 7E**) could potentially hinder the transamination reaction, because the loop movement might bring the ϵ-amino group of PMP into closer proximity to other amino-acid side groups, such as His192, promoting the hydrogen bond formation that may influence the reactivity of PMP with GSA. Such changes could reduce the transamination activity, as suggested by an interference with the formation of diaminovalerate, a proposed metabolic intermediate in this catalytic reaction of GSAAT (Smith et al., 1991). We are aware that this is a hypothesis for which there is currently no direct biophysical confirmation.

In light of experimental evidence showing the different monomeric and dimeric states of the Cys substitution mutants compared to GSAAT(WT), we propose that Cys168 and Cys190 are cruical for the protein activity and likely function as redox-active residues. The single mutants GSAAT(C168S), GSAAT(C190S) and the double mutant GSAAT(C168S/C190S) all showed decreased redox-induced dimerization, suggesting a potential formation of an intramolcular disulfide bridge. C396S substitution disables GSAAT homodimerization. It remained to be determined whether intermolecular bonding to another protein, such as GBP, TTP1 or ALAD (Sinha et al., 2022, Herbst et al., 2023, Wittmann et al., 2024) would stabilize the dimeric GSAAT in the ALA synthesizing complex.

## Data Availability

The original contributions presented in the study are included in the article/**Supplementary Material**. Further inquiries can be directed to the corresponding author.

## Supplementary material

Supplementary Figure 1 Structural insights of GSAAT protein.

Supplementary Figure 2 Redox-dependent structural changes of recombinant GSAAT.

Supplementary Figure 3 Characterization of three-week-old gsa2 lines expressing the GSAAT Cys→Ser substitution mutant.

Supplementary Table 1 List of primer.
