# Supplementary material for: Redox regulation of glutamate-1-semialdehyde aminotransferase modulates the synthesis of 5-aminolevulinic acid in Arabidopsis
Source: Front Plant Sci. 2025 Nov 21;16:1645191. doi: 10.3389/fpls.2025.1645191 (PMC12678314; doi:10.3389/fpls.2025.1645191)
Supplement: Supplementary Figure 1 — Structural insights of GSAAT protein. [file DataSheet1.pdf]

Supplementary Figure 1

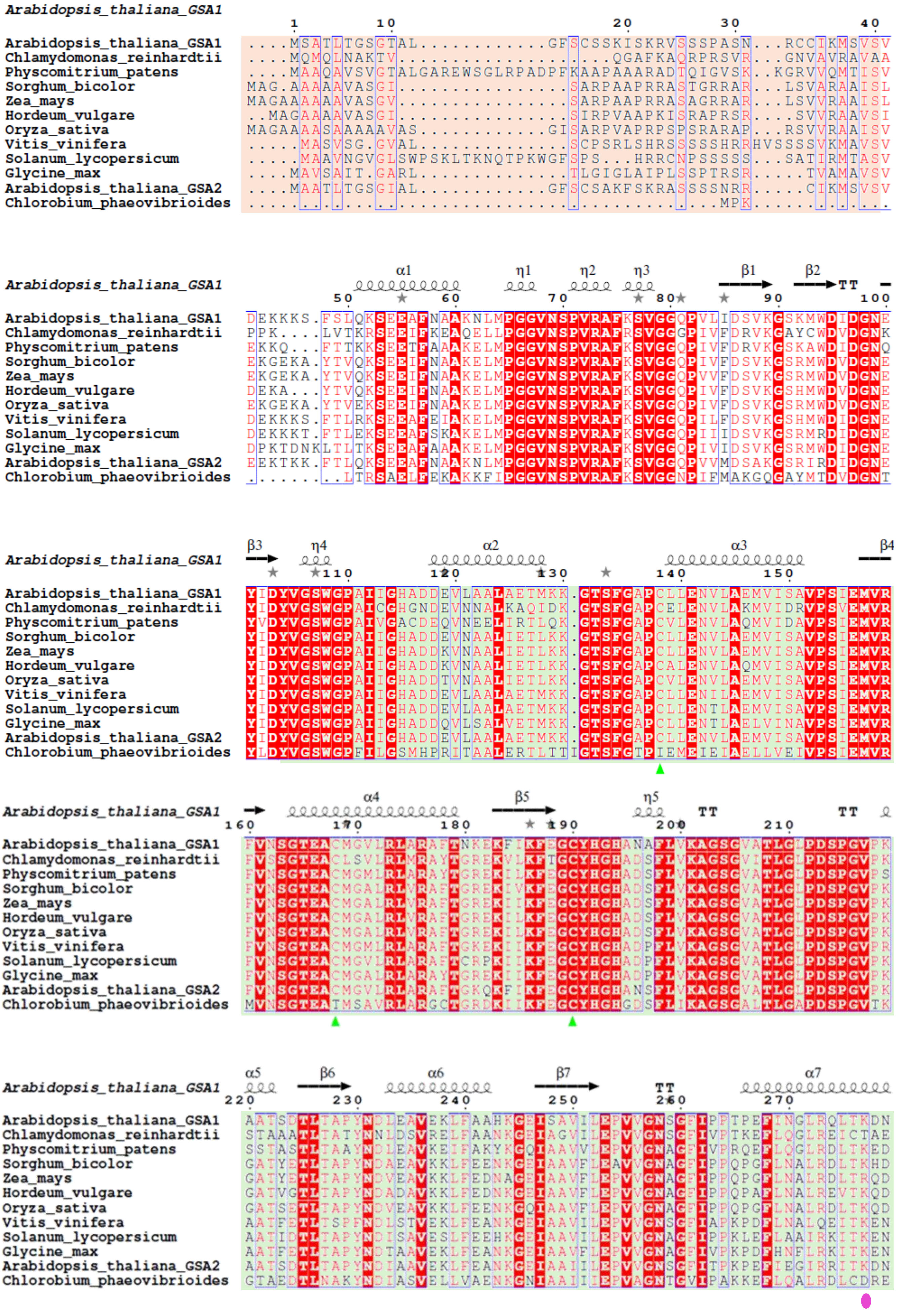

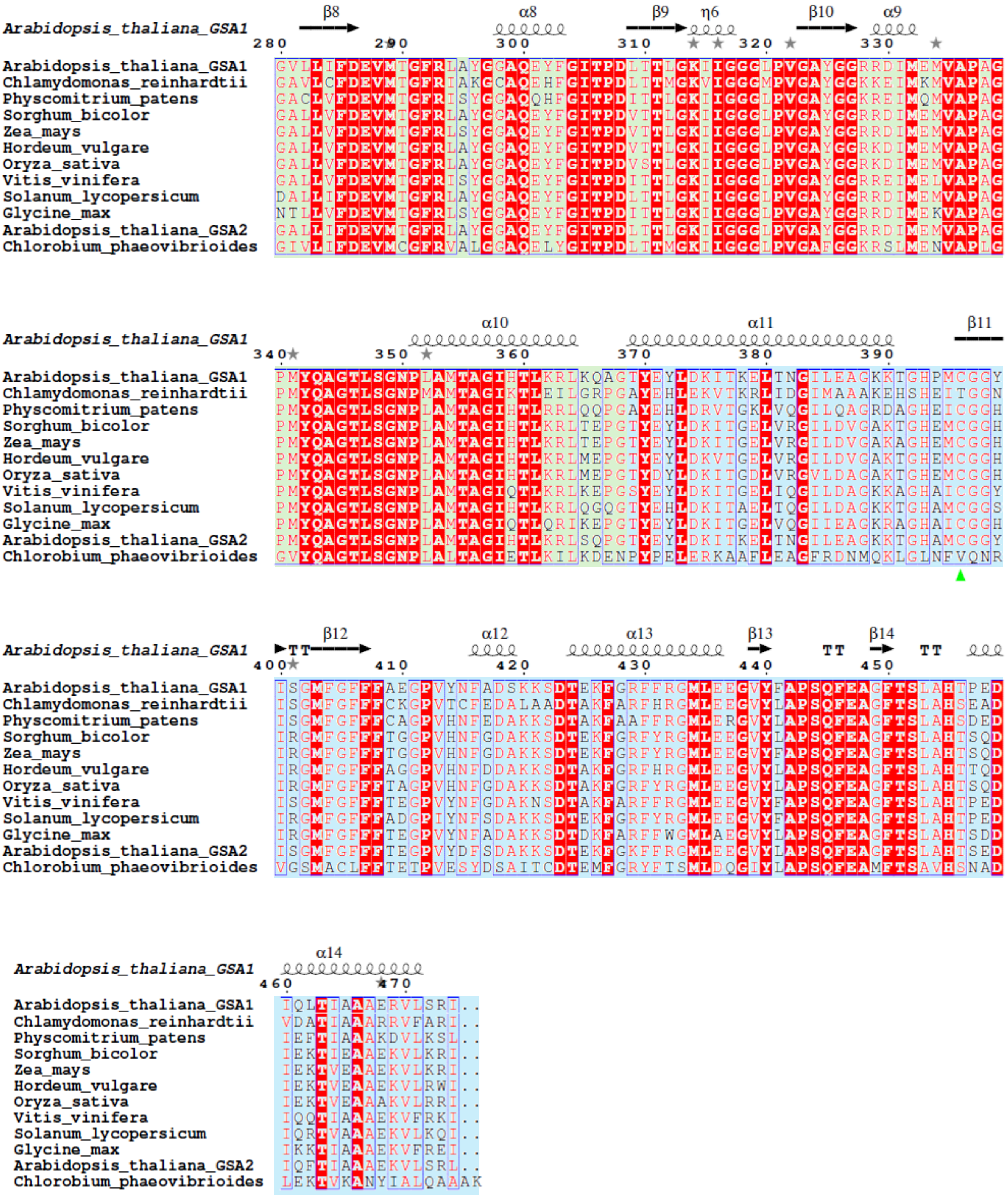

Supplementary Figure 1: Structural insights of GSAAT protein. Alignment of GSAAT of different species including *Arabidopsis thaliana* encoded by *GSA1, GSA2*; *Chlamydomonas reinhardtii, Sorghum bicolor, Zea mays, Hordeum vulgare, Oryza sativa, Vitis vinifera, Solanum lycopersium, Glycine max and Chloribium pheovibrioides*. The four conserved cysteines in GSA proteins of different organisms are highlighted with green arrow heads, while the catalytic lysine residue 274 (K274) is indicated by a pink oval. The multiple sequence alignment was created by MUSCLE, while the secondary structure prediction of GSAAT was performed and visualized in ESPript software.

Supplementary Figure 2

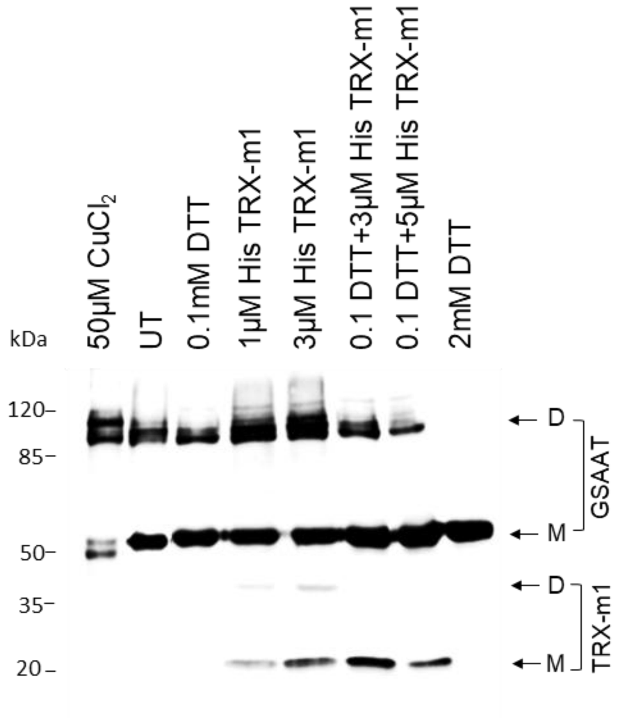

Supplementary Figure 2: Redox-dependent structural changes of recombinant GSAAT. Formation of monomeric and dimeric GSAAT and its redox state under oxidized (50 mM $CuCl_2$), untreated (UT), reduced conditions (0.1 mM DTT, HisTRX-m1 with and without 0.1 mM DTT). The samples were separated by a 10% non-reducing SDS-polyacrylamide gel. The detection of the protein bands was carried out after western blot transfer via a His-tag-specific antibody. The black arrows indicate the presence of a monomer (M) and dimer (D) of proteins.

Supplementary Data, Fig. S3

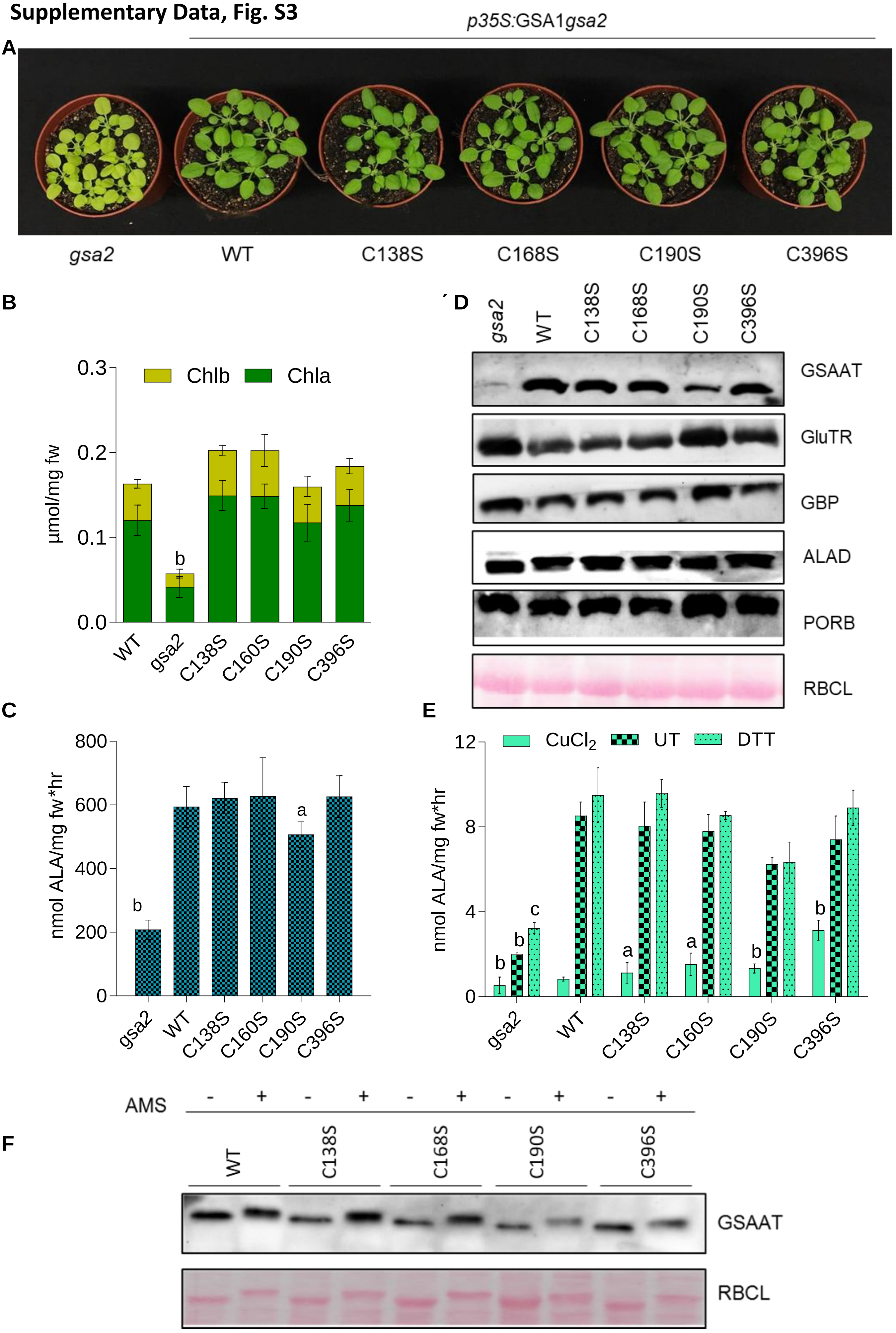

Supplementary Figure 3: Characterization of three-week-old *gsa2* lines expressing the GSAAT Cys→Ser substitution mutant under short-day conditions (SD, 120 µmol photons m^{-2} s^{-1}). **(A)** Images of representative seedlings of homozygous *gsa2* mutants expressing either GSAAT(WT) or one of the GSAAT Cys substitution mutants (GSAAT(C138S), GSAAT(C168S); GSAAT(C190S) or GSAAT(C1396S). **(B)** Comparison of their Chla and Chlb contents. **(C)** ALA-synthesizing capacity of 4-week-old seedlings in detached leaves. ALA, 5-aminolevulinic acid. **(D)** Western blot analysis of *gsa2* complementation lines for different TBS proteins. The Ponceau-stained large subunit of RuBisCO (RBCL) served as a loading control. ALAD, ALA dehydratase; CPOX, coproporphyrinogen oxidase; FC2, ferrochelatase; FLU, Fluorescent in Blue; GBP, GluTR-binding protein; GSAAT, glutamate 1-semialdehyde aminotransferase. GluTR, glutamyl-tRNA reductase; GUN4, GENOMES UNCOUPLED 4; PORB, protochlorophyllide oxidoreductase. **(E)** GSAAT activity **(F)** Immunoanalysis of the AMS-treated transgenic GSAAT variants expressed in the *gsa2* background (GSAAT(WT) and the Cys→Ser GSAAT substitution mutants, which were assayed in leaf extracts after harvesting leaf materials 30 mins after illumination (seedling growth under standard condition). The proteins were separated on 10% non-reducing SDS-PA gels and immunodetected with a GSAAT antibody. Data are given as means and standard deviations of three biological replicates. Statistical significance compared with Col-0 seedlings is indicated by (a) $P \leq 0.05$, (b) $P \leq 0.01$, (c) $P \leq 0.001$ using Student's t-test, fw fresh weight.

Supplementary Figure 4

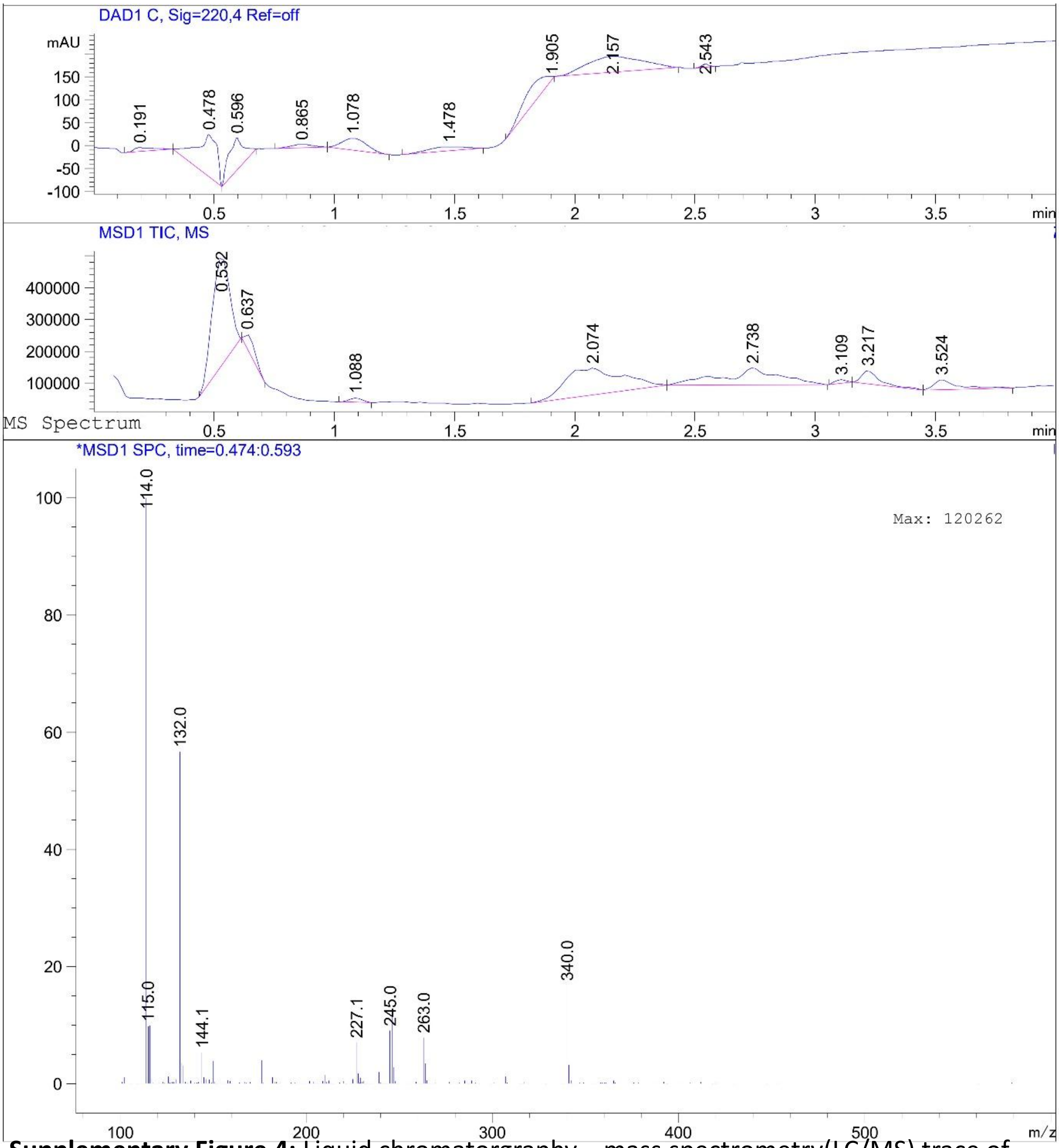

Supplementary Figure 4: Liquid chromatorgraphy – mass spectrometry(LC/MS) trace of glutamate 1-semialdehyde(4-amino-5-oxopentanoic acid. UV/Vis Absorption at 220 nm and total ion chromatogram (TIC) as well as mass spectra for the peak at Rt 0.479-0.593 min. Mass spectrometry analysis was performed with an Agilent Technologies 6120 Quadrupole LC/MS linked to Agilent Technologies HPLC 1290 Infinity using: Thermo Accuore Column RP-MS; Particle Size: 2.6 µm; Dimension: 30 x 2.1 mm; Eluent A: Water with 0.1 % trifluoroacetic acid (TFA); Eluent B: acetonitrile. Flow rate: 0.8 ml/min; UV-detection: 220 nm. MSD, Mass Spectrometric Detector; DAD, Diode Array Detector.
