# Supplementary material for: Redox regulation of glutamate-1-semialdehyde aminotransferase modulates the synthesis of 5-aminolevulinic acid in Arabidopsis
Source: Front Plant Sci. 2025 Nov 21;16:1645191. doi: 10.3389/fpls.2025.1645191 (PMC12678314; doi:10.3389/fpls.2025.1645191)
Supplement: Supplementary Table 1 — List of primer. [file DataSheet2.pdf]

Supplementary Table 1

List of primers

Construct	Primer Sequence (5'-3')
**6X His-protein expression**	
GSA1-Fw	CCCGGGACTTGACTTTCGTATTCGGGT
GSA1-Rv	CCCGGGTAATCGGAAAGGCAAATCGGT
GSA1 (C138S)-Fw	GCTTTGGTGCTCCTAGTCTCTTGGAGAATG
GSA1 (C138S)-Rv	CATTCTCCAAGAGACTAGGAGCACCAAAGC
GSA1 (C168S) -Fw	GGTACTGAAGCGTCTATGGGTGTGCTTC
GSA1 (C168S) -Rv	GAAGCACACCCATAGACGCTTCAGTACC
GSA1 (C190S) -Fw	CATTAAGTTTGAAGGGAGTTATCACGGTCATGC
GSA1 (C190S) -Rv	GCATGACCGTGATAACTCCCTTCAAACTTAATG
GSA1 (C396S)-Fw	CGGGCATCCAATGTCCGGGGGTTACATAAG
GSA1 (C396S)-Rv	CTTATGTAACCCCCGGACATTGGATGCCCG
TRX-f1-Fw	CATATGTGTAGCTTAGAAACCGTTAATG
TRX-f1-Rv	GAGCTCTCATCCGGAAGCAGCAGACCT
TRX-m1-Fw	CATATGCTATCTTCACTCTCGAAGAA
TRX-m1-Rv	AAGCTTTTACAAGAATTTGTTGATGC
NTRC-Fw	CGGGATCCATGGCCACCGCCAATTCTC
NTRC-Rv	CCAGTCGACTCATTTATTGGCCTCAATGAA
**BIFC Analysis**	
GSA1-Fw	CAAAAAAGCAGGCTGATACTAGTACAATGTC
GSA1-Rv	CAAGAAAGCTGGGTGTCTCGAGGATCCTACTCA
TRX-f1-Fw	CAAAAAAGCAGGCTGAATGCCTCTTTCTCTCCGTCTT
TRX-f1-Rv	CAAGAAAGCTGGGTGCCGCTTCTCGCCGCACAAAC
TRX-f2-Fw	CAAAAAAGCAGGCTGAATGCCTCTCTCTCTCCGACT
TRX-f2-Rv	CAAGAAAGCTGGGTGGCCTGACCTTGCTGCTTCAA
TRX-m1-Fw	CAAAAAAGCAGGCTGAATGGCTGCTTACACGTGTACTT
TRX-m1-Rv	CAAGAAAGCTGGGTGCAAGAATTTGTTGATGCTGG
PPOX 2-Fw	CAAAAAAGCAGGCTGATACTAGTACAATGTCGGCGACGC
PPOX 2-Rv	CAAGAAAGCTGGGTGTCTCGAGCTTGTAAGCGTACCGTG
**qPCR Analysis**	
HEMA1-Fw	TTGCTGCCAACAAAGAAGAC
HEMA1-Rv	CCGTCTCCAATGAATCCCTC
GSA1-Fw	TCAAAGAAGAGCGACACAGAG
GSA1-Rv	GTAAACACCTTCTTCCAACATTCC
GSA2-Fw	GAATCACACCTGACTTAACAACTC
GSA2-Rv	GATGTCTCTTCTTCCACCGT
GBP-Fw	ATCTAGACTTTGTGGTTTCAGAAA
GBP-Rv	TGGAAATGGAATCCTCACATC
UROD-Fw	GACCGAAGAAATTGAAAGAGTTGTG
UROD-Rv	ACAAACTCACCCAAACCTACC
CPOX-Fw	GAAGACAGGAGGACGAATAGAG
CPOX-Rv	GATACAGTGATAAGTTGTCGCC
FC2-Fw	CCATACGTTGGTGCTATGGCT

FC2-Rv	CGAGCGGAACTAACGACTGTC
ClpC1-Fw	CAATCGACTCCACCGTCTTT
ClpC1-Rv	TCCCATTTTCCCTTGCATTCT
TTP1-Fw	TCAGGTTGAGTTACTTCTCGG
TTP1-Rv	TATAATTCCCTTAGCCAAGTAGCC
PORB-Fw	TGATTACCCTTCAAAGCGTCTCA
PORB-Rv	CAATGTATTCGTGTTCCCGGT
